# Identification of Prognostic Model and Biomarkers for Cancer Stem Cell Characteristics in Glioblastoma by Network Analysis of Multi-Omics Data and Stemness Indices

**DOI:** 10.3389/fcell.2020.558961

**Published:** 2020-10-19

**Authors:** Jianyang Du, Xiuwei Yan, Shan Mi, Yuan Li, Hang Ji, Kuiyuan Hou, Shuai Ma, Yixu Ba, Peng Zhou, Lei Chen, Rui Xie, Shaoshan Hu

**Affiliations:** ^1^Department of Neurosurgery, The Second Affiliated Hospital of Harbin Medical University, Harbin, China; ^2^Translational Medicine Research and Cooperation Center of Northern China, Heilongjiang Academy of Medical Sciences, Harbin, China; ^3^Department of Pharmacology (The State-Province Key Laboratories of Biomedicine-Pharmaceutics of China, Key Laboratory of Cardiovascular Research, Ministry of Education), College of Pharmacy, Harbin Medical University, Harbin, China; ^4^Department of Neurosurgery, The First Affiliated Hospital of Harbin, Harbin, China; ^5^Department of Digestive Internal Medicine, Harbin Medical University Cancer Hospital, Harbin, China

**Keywords:** connectivity map, machine learning methods, glioblastoma, prognostic model, stemness, tumor immune environment

## Abstract

The progression of most human cancers mainly involves the gradual accumulation of the loss of differentiated phenotypes and the sequential acquisition of progenitor and stem cell-like features. Glioblastoma multiforme (GBM) stem cells (GSCs), characterized by self-renewal and therapeutic resistance, play vital roles in GBM. However, a comprehensive understanding of GBM stemness remains elusive. Two stemness indices, mRNAsi and EREG-mRNAsi, were employed to comprehensively analyze GBM stemness. We observed that mRNAsi was significantly related to multi-omics parameters (such as mutant status, sample type, transcriptomics, and molecular subtype). Moreover, potential mechanisms and candidate compounds targeting the GBM stemness signature were illuminated. By combining weighted gene co-expression network analysis with differential analysis, we obtained 18 stemness-related genes, 10 of which were significantly related to survival. Moreover, we obtained a prediction model from both two independent cancer databases that was not only an independent clinical outcome predictor but could also accurately predict the clinical parameters of GBM. Survival analysis and experimental data confirmed that the five hub genes (CHI3L2, FSTL3, RPA3, RRM2, and YTHDF2) could be used as markers for poor prognosis of GBM. Mechanistically, the effect of inhibiting the proliferation of GSCs was attributed to the reduction of the ratio of CD133 and the suppression of the invasiveness of GSCs. The results based on an *in vivo* xenograft model are consistent with the finding that knockdown of the hub gene inhibits the growth of GSCs *in vitro*. Our approach could be applied to facilitate the development of objective diagnostic and targeted treatment tools to quantify cancer stemness in clinical tumors, and perhaps lead considerable benefits that could predict tumor prognosis, identify new stemness-related targets and targeted therapies, or improve targeted therapy sensitivity. The five genes identified in this study are expected to be the targets of GBM stem cell therapy.

## Introduction

Glioblastoma multiforme (GBM) is the most commonly diagnosed and devastating primary tumor in the central nervous system in adults. Various therapies have been broadly used and have significantly improved the survival of GBM patients, but the median survival of GBM patients is less than 15 months after a definite diagnosis (Ohgaki and Kleihues, 2005; Verhaak et al., 2010). Therefore, it is necessary to make breakthroughs to cure GBM radically.

Stemness, considered to be the capacity to self-renew and differentiate from primordial cells, was initially attributed to the ability of normal stem cells to induce all cell types in an adult organism. GBM stem cells (GSCs), as a population of cancer stem cells with the remarkable ability to promote tumor cell invasion and growth, enhance the tolerance of GBM cells to radiation and chemotherapy. Additionally, frequent aberrations in the transcription and epigenetics of cancer cells often lead to cancerous dedifferentiation and the acquisition of stem cell characteristics by altering the core signaling pathways of normal stem cells (Young, 2011; Bradner et al., 2017). GBM with epigenetically distinct cancer stem cell-like cells (CSCs) often possessed heterologous and endogenous characteristics (Staberg et al., 2018). However, a comprehensive understanding of GBM stemness remains elusive.

In this study, we aimed to perform an integrated analysis of multi-omics data to determine the diagnosis and therapeutic benefits of the stemness of GBM. Two independent stem indices were utilized to comprehensively analyze the stemness characteristics of GBM to determine its diagnosis and prognosis value, which was determined by epigenetic data using a one-class logistic regression (OCLR) machine learning algorithm. One (mRNAsi) was reflective of gene expression, and the other was the epigenetically regulated mRNAsi (EREG-mRNAsi), which was generated using a set of epigenetic regulatory genes associated with stemness.

The association was first examined between mRNAsi and clinicopathological parameters and designated molecular markers that might help guide the prognosis prediction of GBM patients (*n* = 174). We obtained a preliminary understanding of the interaction between stemness and the immune infiltration profiled by the ESTIMATE algorithm (Newman et al., 2015). Besides, we retrieved 55 potential compounds that target the pathways associated with GBM stemness by using the Connectivity Map (CMap) database (Subramanian et al., 2017). Following, by combining weighted gene co-expression network analysis (WGCNA) with differential analysis, we identified 18 stemness-related genes, 10 of which were significantly related to survival. Moreover, we employed a machine learning approach and obtained a prediction model from The Cancer Genome Atlas (TCGA) database and validated in the Chinese Glioma Genome Atlas (CGGA) database. Importantly, this model was not only an independent clinical outcome predictor but could also accurately predict the clinical parameters of GBM. Finally, five stemness-related biomarkers (CHI3L2, FSTL3, RPA3, RRM2, and YTHDF2) were identified by survival analysis and retrospective clinical studies, and further *in vivo* experiments elaborated their key roles in stem cell proliferation and invasion.

## Materials and Methods

### Data Collection and Processing

The RNA-sequencing profile data with corresponding clinical annotation and masked copy number segment of 169 GBM samples (156 Primary and 13 Recurrent) and five healthy samples were obtained from the TCGA database^1^. Two batches of transcriptome data were downloaded from the CGGA database^2^. The “SVA” package was used to integrate the microarray data and decrease heterogeneity between the two batches (Leek et al., 2012). The “normalizeBetweenArrays” function of the “limma” package was used to normalize the transcriptome expression profiles to remove the inter-batch effects. Finally, 388 GBM samples were obtained from the CGGA database. The clinical information corresponding to GBM samples from the TCGA and CGGA datasets was summarized in Supplementary Table S1. These data were updated as of September 26, 2019. Next, the Ensemble ID was converted to the gene symbol matrix by the convert script in Perl^3^.

We also downloaded the GBM (GSCs) microarray dataset (GSE22866 and GSE124145) from the Gene Expression Omnibus (GEO) database. After annotating the data, log2 transformation and normalization of the expression values were performed through the “limma” R package.

### Evaluation of the Associations Between the Stemness Index and Clinical Outcomes in GBM

MRNAsi is an OCLR-based stemness index derived from transcriptomic data. EREG-mRNAsi was derived from a new set of signatures using the OCLR for each molecular feature. Both of these indicators were taken from supporting information in published papers (Malta et al., 2018). The stemness index (mRNAsi), defined as a single continuous covariate, were classified into two subgroups by the median cutoff value. Kaplan-Meier (K-M) analysis was then performed to compare the overall survival (OS) between the two subgroups with the log-rank test. The adjusted *P*-value for multiple testing was applied by the Benjamini-Hochberg (BH) method to explore the association between the index and age, sample type, the status of isocitrate dehydrogenase (IDH), and cytosine-phosphate-guanine island methylator phenotype (G-CIMP). Age (continuous variable) was stratified by the median value.

### Correlation Between GBM Stemness and Immunity

ESTIMATE, as a new algorithm based on gene expression signatures, was applied to assess the fraction of stromal cells and the infiltration of immune cells in the tumor samples (Yoshihara et al., 2013). ESTIMATE scores represent tumor purity. We calculated the proportion of immune cells for each given GBM sample using a *P*-value < 0.05 as the screening criterion. For any of the selected GBM samples, we calculated the association between mRNAsi and the relative proportion of immune and stromal cells. The *P*-values of the relevance were calculated using the Pearson test.

### Single-Sample Gene-Set Enrichment Analysis (ssGSEA)

Single-sample gene-set enrichment analysis (ssGSEA) was employed to quantify the relative enrichment of each immune cell fraction with the gene sets (Hanzelmann et al., 2013). The ssGSEA score was normalized to a percentile distribution, where 0 was the minimum value of immune cell abundance and 1 was the maximum value. To discover the underlying mechanism of different subgroups, typical biological processes, including (1) Angiogenesis; (2) antigen processing machinery; (3) CD8 T-effector signature; (4) cell cycle; (5) DNA damage repair; (6) DNA replication; (7) epithelial-mesenchymal transition (EMT) markers including EMT1, EMT2, and EMT3; pan-fibroblast (8) FGFR3-related genes; (9) Immune checkpoint; (10) Mismatch repair; (11) Nucleotide excision repair; (12) TGF-β response signature (Pan-F-TBRS); (13) WNT targets was quantified by ssGSEA with a list of gene sets (Mariathasan et al., 2018). Nine gene sets of oncogenic pathways were also introduced into our analysis to explore the mechanism of regulation on different subclasses (Sanchez-Vega et al., 2018).

### Selection of Differentially Expressed Genes (DEGs)

The “limma” package in R was used to investigate the transcriptome data to identify differentially expressed genes (DEGs) between the high and low subgroups (Ritchie et al., 2015). The selection criteria were as follows: false discovery rate (FDR) < 0.05 and | log_2_ fold change| > 2 (Cheng et al., 2019). The expression value of the same-named gene was averaged, and a gene with an average value of >0.2 was selected as a research gene.

### Functional Enrichment Analysis

The Gene Ontology (GO) and Kyoto Encyclopedia of Genes and Genomes (KEGG) pathway analyses were applied to annotated the functions of the selected DEGs using the “clusterProfiler” package in R (Wilkerson and Hayes, 2010). The terms of GO and KEGG with *q*-value < 0.05 were filtered as significant functions. The gene set enrichment analysis (GSEA) is a computational methodology utilized to examine whether a set of designated genes possess significant and consistent deviation between various parameters. In this study, GSEA (default parameters) was employed to uncover the hallmarks. | NES| > 1, normalized *P*-value < 0.05 and FDR *q*-value < 0.25 were considered as statistically significant described as previous study (Subramanian et al., 2005).

### WGCNA and Module Preservation

The R package called “WGCNA” was used to perform WGCNA (Langfelder and Horvath, 2008). DEGs with no apparent fold change may be significantly associated with co-expressed gene modules in WGCNA. The genes in the same module indicate that their functions and regulations are related. Genes with significant differences were screened to provide heterogeneity and accuracy assurance for the bioinformatics statistics. We constructed a weighted network to calculate the adjacency capacities using the soft thresholding power parameter. Next, a topological overlap matrix (TOM) transformed from the adjacency matrix was obtained. Consensus TOM was defined as the input for hierarchical clustering, and modules were identified using the Dynamic Tree Cut algorithm with minModuleSize = 50. Modules with similar expression proles were merged with the merging threshold of 0.25.

### Confirmation of Significant Modules

To further explore the relationship between the two indices and gene expression, we define the two indices as clinical phenotypes. The overlaps of phenotypes and consensus modules were calculated with the hypergeometric test, and the *P*-value was assigned as module membership (MM). A color-coded table of the *P*-values was created. Gene significance (GS) was defined as the correlation between the expression and the phenotype. The key genes of the most relevant modules were selected with the threshold MM > 0.8 and GS > 0.5.

### Hub Gene Identification and Further Analysis

The intersection of DEGs based on EREG-mRNAsi grouping and mRNAsi grouping and the key genes was screened out as hub genes for subsequent analysis. The Venn diagram was drawn with the website tool.

The Oncomine^4^ database was employed to detect differences with the default retrieval threshold. An interaction network was produced by the STRING^5^ database and was reconstructed via Cytoscape software (Shannon et al., 2003; Franceschini et al., 2013).

### Confirmation and Validation of the Prognostic Value of the Hub Genes

Univariate Cox was performed to detect the prognostic value of the selected hub genes. Genes whose *P*-values were less than 0.3 were selected to build a potential predictive model through the LASSO-penalized Cox regression algorithm.

Subsampling was applied from the training set in a 7:3 ratio with 1,000 cross-validations (Tibshirani, 1997). Finally, seven genes with their regression coefficients were obtained. The risk score was calculated using the formula:

$$\begin{array}{c} \mathrm{Risk}\mathrm{score}=\sum_{i=1}^{n}\mathrm{Coef}\times \exp \end{array}$$

where Coef is the coefficient, and exp is the expression value. To explore whether the predictive capacity of the risk model could independently predict other clinical parameters of GBM (including age, sex, molecular subtype, and gene mutation), we performed a multivariate Cox analysis of the patients. Samples were dichotomized into low or high expression groups based on the median risk score. The OS of both groups was compared using the K-M method and the log-rank test. Chi-square tests were used to measure the distribution of gender, GBM subtype, IDH1 status, ATRX status, G-CIMP status, age, and sample type between the two risk groups. The area under the curve (AUC) of the receiver operating characteristic (ROC) curve was used to estimate the prediction accuracy of the model. All algorithms and methods were equally applied during the verification process in CGGA.

### Identification of Potential Compounds

Connectivity Map (updated in September 2017)^6^, was employed to search for compounds that might target GBM stemness-related pathways with the default cutoff value (Subramanian et al., 2017).

### Immunohistochemistry

Tissue microarray chips containing 85 GBM and 15 brain tissue were obtained from Outdo Biotech (Shanghai, China). Immunohistochemistry (IHC) staining was performed as previously described (Cheng et al., 2019). Histochemistry Score was calculated with the formula with the Quant Center Analysis tool:

$$\begin{array}{c} \mathrm{Histochemistry}\mathrm{score}=\sum_{i=1}^{n}\mathrm{PI}\times i \end{array}$$

where PI is the percentage of cells in various intensity with corresponding coefficients (i) (Azim et al., 2015).

### Cell Culture and Transient Transfection

The GSC lines NCH64436 were purchased from Cell Line Services (Eppelheim, Germany), and cell culture was performed according to the instructions. NCH64436 cells were seeded in six-well plates and transfected with plasmid by Lipofectamine 2000 (Invitrogen, United States) based on the instructions. Target sequences for shRNAs were summarized in Supplementary Table S2.

### RNA Extraction, RT-PCR and qRT-PCR

Total RNA extracted from transfected cells was reverse transcribed with RT reagent Kit gDNA Eraser (TaKaRa) and detected by SYBR-Green (TaKaRa). The PCR primers were listed in Supplementary Table S2.

### Transwell Assay, MTT Assay, and Cell Cycle Analysis

Transwell and MTT assay were applied based on the previous method (Huang et al., 2019). CD133 Indirect Isolation kit (Miltenyi Biotec) was used to evaluate the ratio of CD133+ cells by flow cytometry. For TMZ treatment, GSCs were seeded at 4000 cells/well (96-well) treated with increasing concentrations of TMZ (10–400 μm) for 48 h followed by MTT assays.

### Sphere-Forming and Limiting Dilution Assays

To investigate the self-renewal capacity of GSCs, GSC lines NCH64436 were incubated with Accutase, dissociated into single cells, and seeded in 96-well plates with 200 μL/well of stem cell-conditioned medium. The *in vitro* limiting dilution assay was performed as described previously. Briefly, NCH64436 cultured were collected, dissociated into single cells, and seeded in 96-well plates at a density of 5, 10, 20, 50, 100, 200, or 400 cells per well and each well was then examined for formation of tumorspheres after 9 days. Wells without tumorspheres were counted for each group.

### *In vivo* Functional Assay

The *in vivo* assay was performed according to the ethical guidelines for laboratory animal use and approved by the Ethics Committee of Harbin Medical University (SYDW-2019-8-2). For subcutaneous tumor models, approximately 1 × 10^5^ of GSCs in 0.2 mL of PBS were injected subcutaneously into the 4-week-old female nude mice (*n* = 5 mice/group), respectively. Mice were checked every 3 days. After 21 days, mice were sacrificed, tumors were excised, weighed, and photographed.

### Statistical Analysis

R software version 3.6.4 was used for all statistical analyses. The strategies based on a machine learning approach were implemented with the R package “gelnet” with default settings. The ROC curve was drawn by OriginPro software (Ver. 9, OriginLab, Northampton, MA, United States). Correlation analysis between the risk value and clinical parameters was plotted by GraphPad Prism version 8.3 (GraphPad Software, San Diego, CA, United States). *P* < 0.05 was considered statistically significant.

## Results

### Stemness Index in GBM

**FIGURE 1:**
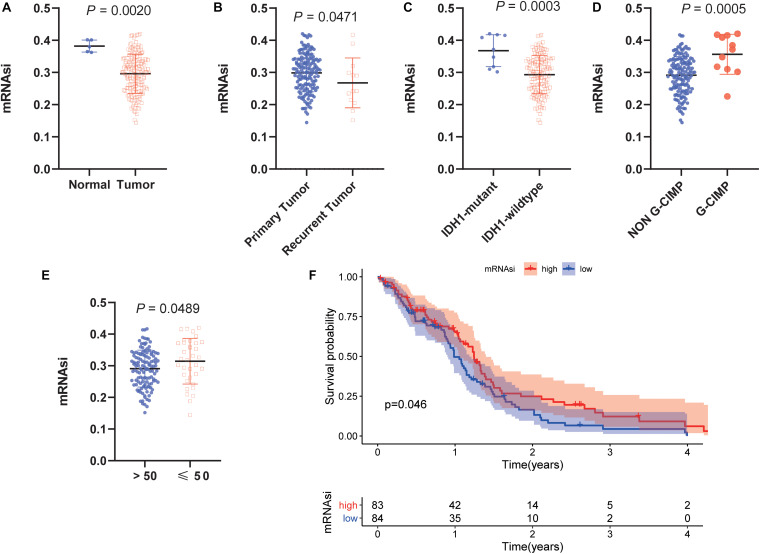
Clinical and molecular features associated with the mRNA expression-based stemness index (mRNAsi) in glioblastoma (GBM). **(A)** Boxplots of mRNAsi in individual samples stratified by sample type. **(B)** Boxplots of mRNAsi in individual samples stratified by primary and recurrent status. **(C)** Boxplots of mRNAsi in individual samples of patients with GBM stratified by isocitrate dehydrogenase 1 (IDH1) status. **(D)** Boxplots of mRNAsi in individual samples of patients with GBM stratified by cytosine-phosphate-guanine island methylator phenotype (G-CIMP) status. **(E)** Boxplots of mRNAsi in individual samples stratified by age. **(F)** Kaplan-Meier (K-M) curves showing the OS of GBM patients with low and high mRNAsi based on the median cutoff point.

To determine the correlation between the molecular/clinical characteristics and the stemness of GBM samples, we performed a differential analysis. MRNAsi, as an indicator to describe the degree of differentiation of GSCs, was applied to this study (Malta et al., 2018). Normal samples had significantly higher mRNAsi values than GBM samples (Figure 1A). For recurrent tumors and primary tumors, mRNAsi showed a higher trend in primary tumors (Figure 1B). Similarly, mRNAsi was significantly related to IDH1 status and G-CIMP status (Figures 1C,D). We also found that samples from patients older than 50 years (median value) of age also showed a significant decrease in mRNAsi value (Figure 1E). To further explore the impact of the index on survival, we performed K-M survival analysis and observed that patients with higher mRNAsi scores had longer OS times than those with lower mRNAsi scores (*P* < 0.05) (Figure 1F). The above results indicate that there is a significant correlation between mRNAsi and the clinical molecular parameters of GBM, and we speculate that the stemness is valuable for the subsequent analysis.

### Association of the Stemness With the Immune Microenvironment of GBM

**FIGURE 2:**
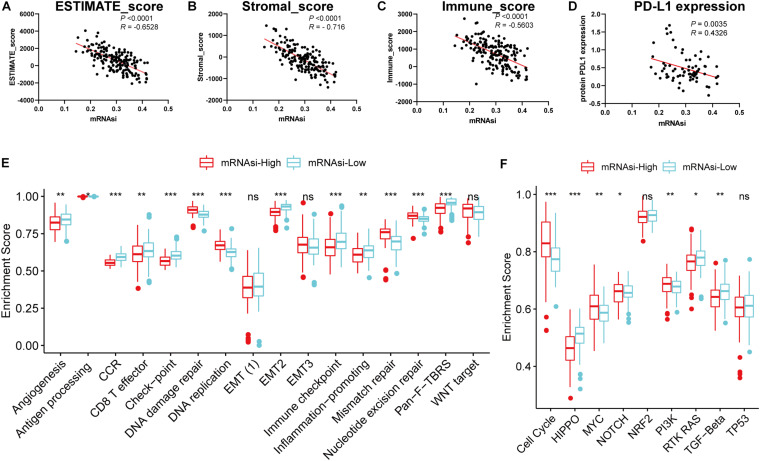
Associations of the stemness index with the immune microenvironment in GBM. **(A–D)** Correlation between mRNAsi and ESTIMATE score **(A)**, stromal score **(B)**, immune score **(C)**, the relative expression of PD-L1 **(D)**. **(E–F)** Differences in immune-related cellular pathways **(E)** and nine oncogenic pathways **(F)** between low and high mRNAsi groups in the TCGA-GBM cohort. The upper and lower ends of the boxes represented an interquartile range of values. The lines in the boxes represented the median value, and the dots showed outliers. The asterisks represented the statistical *P*-value *P < 0.05; **P < 0.01; ***P < 0.001, ns, no significant.

Considering that the powerful correlation between clinical/molecular characteristics and mRNAsi, we measured the relationship between mRNAsi and immune microenvironment. We found that mRNAsi was significantly negatively correlated with tumor purity, the presence of stromal cells and immune cells (Figures 2A–C). Considering that GSCs capable of up-regulating the expression of PD-L1 and promoting the immune escape of GBM cells, we also analyzed them and found that they have a significant negative correlation (Figure 2D; Hsu et al., 2018). In addition, we found differences in immune signaling based on the stratification strategy of stemness. We found that immune-related signaling pathways were highly enriched in the low mRNAsi subgroup, while DNA-related pathways were highly enriched in the high mRNAsi subgroup (Figure 2E). These results are consistent with previous results that immunity and stemness were negatively correlated. Moreover, we also found significant differences in nine canonical oncogenic pathways between the two subgroups (Figure 2F). We thus speculate that as immune cell infiltration or PD-L1 pathway gradually decreases with stemness, GBM is less sensitive to immune checkpoint blocking therapy, making further immunotherapy less effective. Our findings were consistent with previous reports that the central nervous system is an independent immune system and that immunotherapy faces various challenges that need to be resolved urgently (Jackson et al., 2019). Given the significant correlation between the stemness index and GBM tumor immune microenvironment and clinical parameters, the application of mRNAsi for subsequent network analysis is remarkably convincing.

### Determination of Potential Mechanisms and Compounds Related to Stemness

**FIGURE 3:**
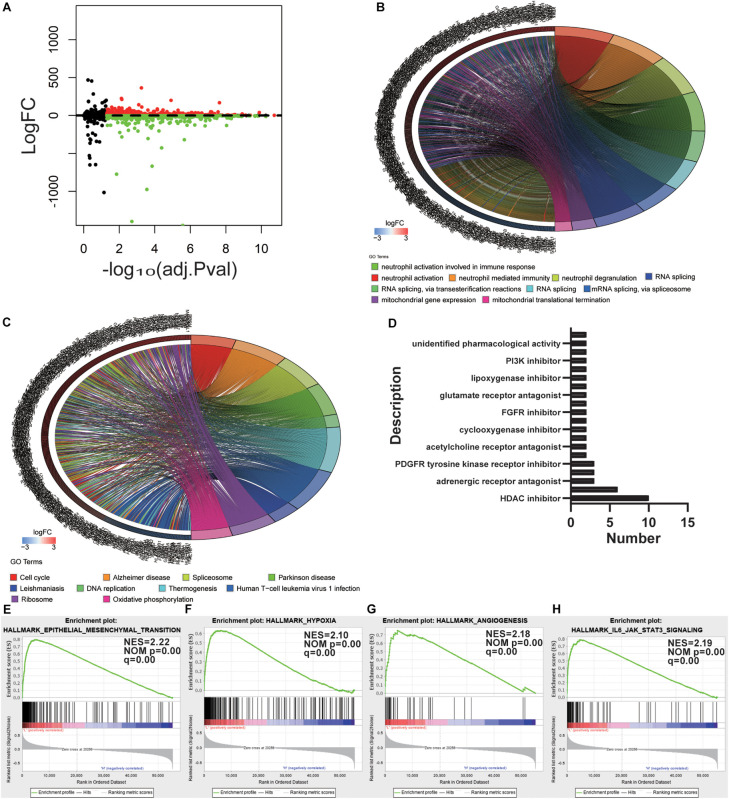
Determination of potential mechanisms related to stemness. **(A)** Volcano plot with high and low mRNAsi grouping difference analysis. **(B,C)** Functional annotation of the upregulated DEGs in the low mRNAsi subgroup of GO analysis **(B)** and KEGG pathway analysis **(C)**. **(D)** Histogram showing the number of compounds in the top 10 MoA, sorted by descending number of compounds with a shared MoA. The above compounds have an enrichment score ≤–95 and might be capable of targeting the GBM stemness signature. **(E–H)** GSEA revealed that genes with higher expression in the low mRNAsi subgroup were enriched for hallmarks of GBM.

To uncover the relevance of the gene expression patterns of GBM with mRNAsi, the samples with GBM were divided into two subgroups according to the median value of mRNAsi. The DEGs were determined by applying the “lmfit” function in R (Ritchie et al., 2015). The volcano plot showed distinct gene expression patterns of patients who belong to high vs. low mRNAsi groups (Figure 3A). For comparison based on mRNAsi, 963 DEGs were down-regulated and 1702 DEGs were up-regulated in the low mRNAsi subgroup (| log_2_ fold change| > 2, FDR < 0.05). We also tested the up-regulated DEGs in the low mRNAsi subgroup, and we uncovered that out of 1702 DEGs, 181 genes were significantly related to poor prognosis (*P* < 0.05, representative figures were shown in Supplementary Figure S1). These findings suggest that the two subgroups are a robust classification.

To outline the intrinsic mechanism of the stemness degree affecting the malignant process of GBM, we performed functional annotation of the designated DEGs. Top GO terms were identified, included neutrophil activation, neutrophil degranulation, RNA splicing, and mitochondrial gene expression (Figure 3B). Also, top KEGG terms mainly included cell cycle, spliceosome, DNA replication, and oxidative phosphorylation (Figure 3C). All these findings uncovered the malignant development-related pathways and processes of stemness in GBM.

To determine the potential compounds that target the pathways associated with GBM stemness, the DEGs based on the mRNAsi grouping were submitted to the CMap database. The top 55 compounds were summarized in Supplementary Table S3 (Figure 3D). In total, 132 mechanisms were revealed through the CMap mode of action (MoA) analysis. Ten compounds (NCH-51, apicidin, trichostatin-a, belinostat, ISOX, vorinostat, entinostat, pyroxamide, valproic acid, and panobinostat) shared the MoA of HDAC inhibitors, and six compounds (belinostat, vorinostat, entinostat, panobinostat, pyroxamide, and pyroxamide) shared the MoA of cell cycle inhibitors. We also found that SU-11652 and ENMD-2076 shared the MoA of VEGFR inhibitors. Recently, various pharmacological studies have shown that compounds that can act on multiple genes or mechanisms should be valued. In this study, the mechanism of action of the different compounds we retrieved was similar, suggesting that specific treatments can produce considerable efficacy against the undifferentiated phenotype of GBM.

Gene set enrichment analysis was also employed to explore the hallmarks in the malignant process of GBM. Four hallmark gene sets were enriched in low mRNAsi subgroup, including epithelial-mesenchymal transition (NES = 2.22, normalized *P*-value = 0.00, *q*-value = 0.00), hypoxia (NES = 2.10, normalized *P*-value = 0.00, *q*-value = 0.00), angiogenesis (NES = 2.18, normalized *P*-value = 0.00, *q*-value = 0.00), and IL6-JAK-STAT3 signaling (NES = 2.19, normalized *P*-value = 0.00, *q*-value = 0.00) (Figures 3E–H). These findings indicate that stemness is involved in the malignant process of GBM.

### Screening of DEGs

**FIGURE 4:**
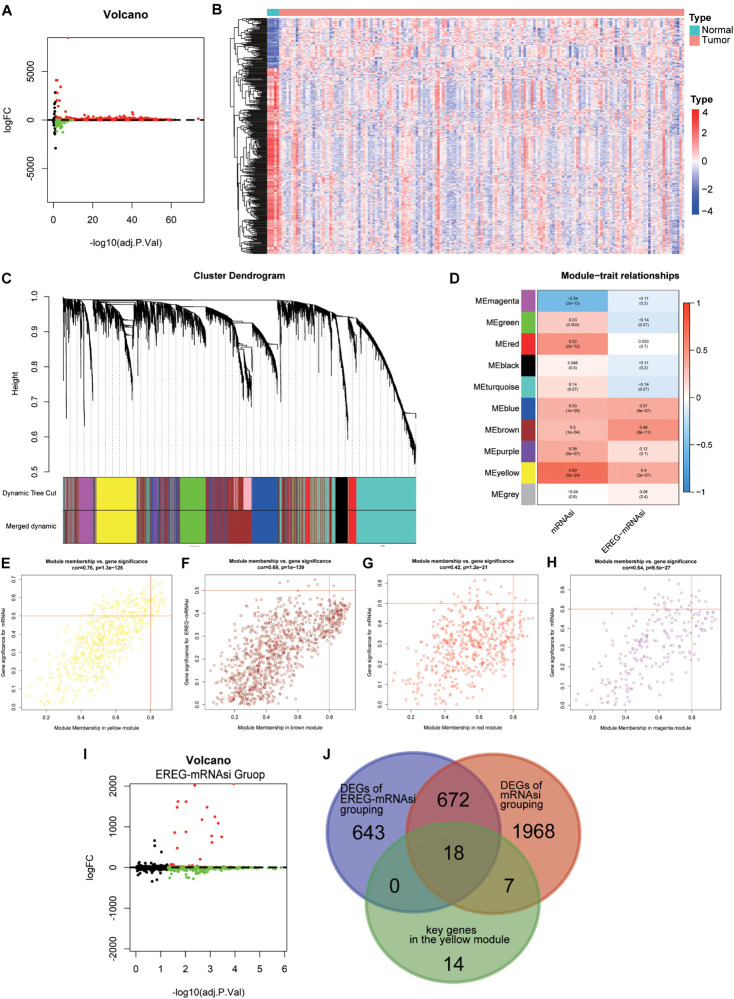
The identification of differentially expressed genes (DEGs) through differential analysis and WGCNA. **(A)** Volcano plot of DEGs (169 GBM samples and five normal samples); red indicates downregulated genes, and blue indicates upregulated genes. **(B)** Heatmap of the expression value of the top 400 median absolute deviations of the differential genes between different sample types. **(C)** Identification of a co-expression module in GBM. The branches of the cluster dendrogram correspond to the 10 different gene modules. Each leaf on the cluster dendrogram corresponds to a gene. **(D)** Correlation between the gene module and clinical traits, including mRNAsi and EREG-mRNAsi. The correlation coefficient in each cell represents the correlation between the gene module and the clinical traits, which decreased in color from red to blue. The corresponding *P*-value is also annotated. **(E–H)** Scatter plot of module eigengenes in the yellow **(E)**, brown **(F)**, red **(G)**, and magenta **(H)** modules. **(I)** Volcano plot of DEGs based on EREG-mRNAsi grouping; red indicates downregulated genes, and blue indicates upregulated genes. **(J)** A Venn diagram showing the overlapping genes among the key genes in the yellow module, DEGs based on mRNAsi grouping and DEGs based on EREG-mRNAsi grouping.

Considering the significant difference in mRNAsi values between normal and tumor tissues, we first screened the DEGs of the tumor and normal tissues through the “limma” package in R (Ritchie et al., 2015). Through this analysis, we obtained 12180 DEGs, of which 7753 were downregulated and 4427 were upregulated (Figures 4A,B). After excluding outlier samples (Supplementary Figure S2A), 5484 DEGs with the cutoff value FDR < 0.05 and | log_2_ fold change| > 2 were placed in a module. To more accurately investigate the genes associated with GBM stemness and determine gene modules with similar expression patterns, we presented a gene scale-free co-expression network via the WGCNA algorithm (Langfelder and Horvath, 2008). The scale-free network is characterized by the existence of a few hub nodes, whose connectivity degree was significantly higher than that of other nodes in this network. To establish the adjacency matrix, we set the soft threshold from 1 to 20 and calculated the optimal beta value with the “pickSoftThreshold” function (Beckerman et al., 2017). Therefore, we selected β = 4 (scale-free *R*^2^ = 0.902) to ensure a scale-free topology and identified 10 modules for subsequent analysis (Figure 4C and Supplementary Figure S2B).

Because of the uniformity of the algorithm for the stemness indices for 33 cancer types and the existence of intra-tumor heterogeneity, it is impossible for mRNAsi to accurately reflect stemness for every cancer type, such as glioma. EREG-mRNAsi, based on RNA expression and epigenetic inheritance, elucidated the differences between epigenetic traits and mRNAsi, although the correlation with clinical traits was not significant. EREG-mRNAsi also reflected, to some extent, the degree of de-differentiation of cancer cells. The application of EREG-mRNAsi could compensate for the above shortcomings, making our study more comprehensive and accurate. The EREG-mRNAsi was considered to be complementary to mRNAsi (Malta et al., 2018). To make our analysis more comprehensive, EREG-mRNAsi derived from the new signature was introduced into the network analysis. The heatmap presented the relationship between the modules and stemness indices with the corresponding *P*-values (Figure 4D). Notably, the correlation of the yellow module and mRNAsi possessed a maximum value of 0.69, followed by 0.54 for the magenta module and mRNAsi, 0.52 for the red module and mRNAsi, and 0.48 for the brown module and EREG-mRNAsi (Figures 4E–H). Finally, we selected the yellow module and the genes in this module for subsequent analysis. Thirty-nine key genes were screened out with the cutoff value MM > 0.8 and GS > 0.5. To elucidate the function of the key genes, GO and KEGG analyses were performed and showed that the dominant functions of this module were nuclear division, organelle fission, and chromosome segregation, which were mainly involved in the cell cycle pathway (Supplementary Figure S3).

Based on the strong correlation between the two stemness indices in the network analysis, we divided the GBM sample into two groups according to the median value of the indices. Differential analyses were performed to compare the low and high stemness indices groups. For the mRNAsi grouping, 2665 DEGs were obtained with the criteria FDR < 0.05 and | log_2_ fold change| > 2 (Figure 3A). For the EREG-mRNAsi grouping, 1333 DEGs were obtained with the same criteria, of which 1019 DEGs were downregulated and 314 DEGs were upregulated (Figure 4I). As shown in the Venn diagram, 18 hub genes were obtained among the DEGs based on mRNAsi grouping, the DEGs based on EREG-mRNAsi grouping, and the key genes in the yellow module (Figure 4J). The intersection of these three genes allows not only a more comprehensive analysis of stemness but also a more precise and accurate identification of the genes associated with stemness.

### Analysis and Validation of Hub Gene Expression

**FIGURE 5:**
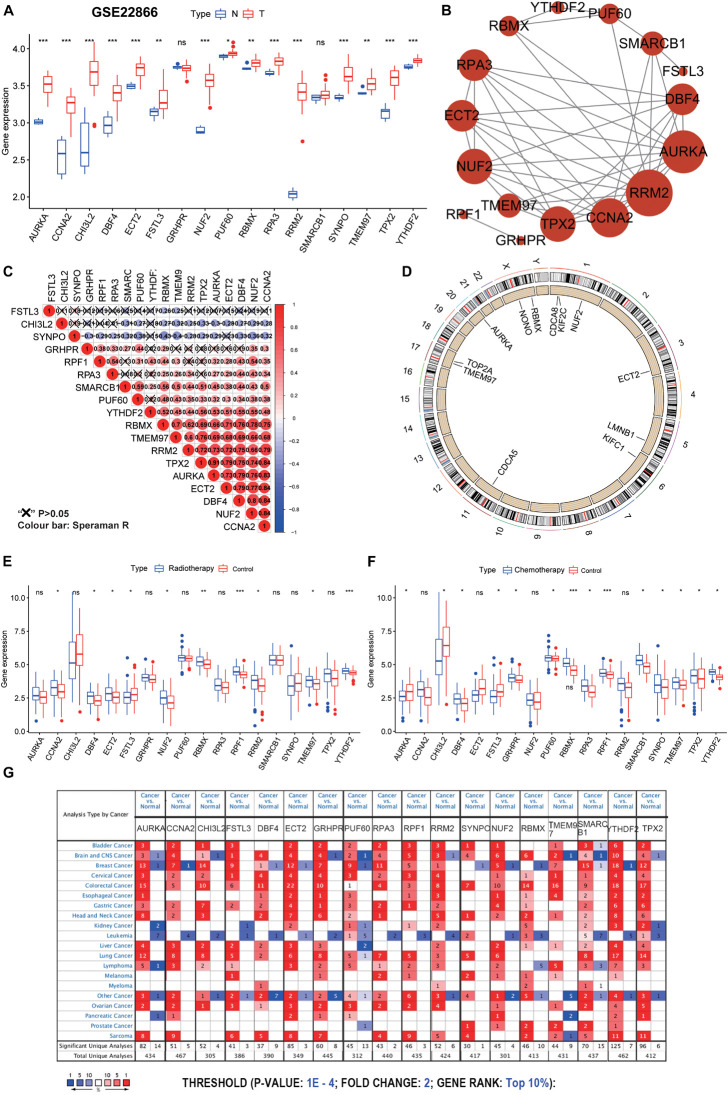
Further validation and analysis of hub genes. **(A)** The 17 hub genes were verified in the GEO database. In GSE22866, the gene expression value of hub genes was higher in GBM (40 samples) than that in normal (five samples). Notably, RRF1 was not found in the GSE22866. **(B)** The protein-protein interactions between the hub genes. The size of the node indicates the number of interacting proteins with the designated protein. **(C)** Spearman correlation analysis of the 18 hub genes. **(D)** Circle plot of differential CNV of hub genes. The black dot in the outer ring indicates amplification, while the red dot in the inner ring indicates deletion. **(E)** Among the 18 hub genes, 10 genes were significantly different between the radiotherapy and control groups. **(F)** Among the 18 hub genes, 14 genes were significantly different between the chemotherapy and control groups. **(G)** The mRNA expression patterns of the hub genes in overall cancers. The mRNA expression difference between tumors and normal tissues was analyzed in the Oncomine database. The number in the colored cell represents the number of analyses meeting these thresholds. The color depth was determined by the gene rank. The red cells indicate that the mRNA levels of the target genes are higher in tumor tissues than in normal tissues, while the blue cells indicate that the mRNA levels of the target genes are lower in tumor tissues than in normal tissues. ***P < 0.001, **P < 0.01, *P < 0.05, ns, no significance.

To further validate the hub genes, we explored the GEO database and analyzed their expression profiles between tumor and normal samples in the GSE22866 dataset. We found that the hub genes, except GRHPR and SMARCB1, were significantly upregulated in GBM patients (Figure 5A). The above findings suggested that the hub genes were closely correlated with the occurrence of GBM. To better understand the interactions among the hub genes, we also analyzed the correlations and interactions among them. AURKA seems to be the hub node, and its interactions or co-expression with RRM2, CCNA2, TPX2, RPA3, and NUF2 were supported both by experimental evidence and by text mining in the STRING database (Figure 5B; Franceschini et al., 2013). For the correlation networks of these genes, CCNA2 also had a significant correlation with MUF2, DBF4, ECT2, TPX2, and AURKA (*P* < 0.05) (Figure 5C). In the copy number variation (CNV) differential analysis between normal and GBM, we found that there were significant differences in the 12 hub genes, which were CDCA8, KIF2C, NUF2, ECT2, LMNB1, KIFC1, NONO, RBMX, CDCA5, TMEM97, TOP2A, and AURKA (Figure 5D). Among the 18 hub genes, the expression value (normalized) of 17 genes (except LMNB1) was found to be significantly related to the status of CNV (Supplementary Figure S4A). It is worth noting that the amplification of all genes was significantly correlated with high mRNA expression values. These multi-omics results indicate that the 18 hub genes may promote the generation and development of GBM through multiple pathways.

Although GBM resistance is mainly due to the existence of GSCs, the traditional DNA repair system (such as O6-methylguanine-DNA methyltransferase, mismatch repair, and base excision repair) is also the cause of treatment resistance (Jiapaer et al., 2018). Among the 18 hub genes, 10 genes were significantly different between the radiotherapy and control groups, and 14 genes were significantly different between the chemotherapy and control groups (Figures 5E,F). Of the 10 genes with significant differences shown in Figure 5E, one gene was significantly down-regulated and nine genes were significantly up-regulated in the radiotherapy group. Of the 14 genes with significant differences shown in Figure 5F, three genes were significantly down-regulated and 11 genes were significantly up-regulated in the chemotherapy group. We predict that these genes may be valuable therapeutic targets for suppressing the stemness characteristics of GBM. Also, through disease summary analysis using the Oncomine database, we found that these genes were not only upregulated in GBM but also in most other cancers (Figure 5G). These findings established a novel approach for stemness-related genes recognition and illuminating insights into the vital roles of GSC-related genes in GBM.

### Prognostic Value of the 18 Hub Genes and the Construction of a Predictive Model

**FIGURE 6:**
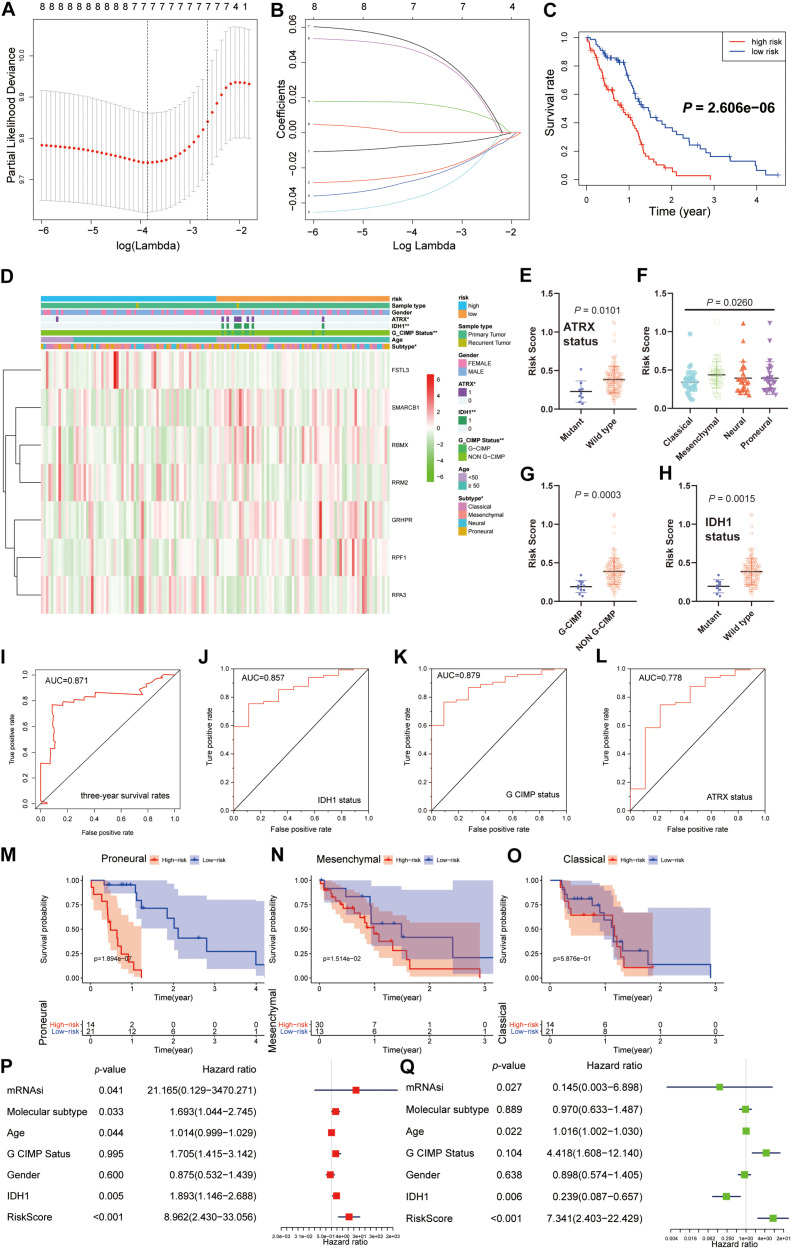
Construction and analysis of the prognostic model based on hub genes from the TCGA dataset. **(A)** Ten-time cross-validation for tuning parameter selection in the LASSO model. **(B)** LASSO coefficient profiles of the eight prognostic genes. **(C)** K-M curves for patients in the TCGA dataset assigned to high- and low-risk groups based on the risk score. **(D)** The heatmap shows the expression levels of the three hub genes in the low- and high-risk GBM groups. The distribution of clinicopathological features was compared between the low- and high-risk groups. **(E–H)** Distribution of risk scores in the TCGA dataset stratified by ATRX status **(E)**, molecular subtype **(F)**, G-CIMP status **(G)**, and IDH status **(H)**. **(I–L)** ROC curves showed the predictive efficiency of the predictive model for the 3-year survival rate **(I)**, IDH1-mutant status **(J)**, G-CIMP status **(K)**, and ATRX-mutant status **(L)**. **(M–O)** K-M curves showing the OS of each subtype of GBM patients with high or low-risk scores based on the median cutoff point. **(P,Q)** Univariate **(P)** and multivariate **(Q)** Cox regression analyses of the association between clinicopathological factors (including the risk scores) and OS of patients in the TCGA datasets. **P < 0.01, *P < 0.01.

We next sought to investigate the prognostic role of the hub genes related to the stemness indices in GBM. We performed univariate analyses for OS by using the hub genes from the GBM cohort. Considering the limited number of genes, nine genes were selected with a cutoff value of *P* < 0.3. To promote the use of the hub genes, clinical predictive models were constructed by the LASSO algorithm based on the optimal lambda value (Figures 6A,B). As shown in Figure 6C, the predictive model performed reasonably well in distinguishing good and poor clinical outcomes in patients with GBM based on median risk score. We observed that the high- and low-risk subgroups possessed significant differences in ATRX status (*P* < 0.05), IDH1 status (*P* < 0.01), G-CIMP status (*P* < 0.01), and molecular subtype (*P* < 0.05) (Figure 6D). We observe that patients with G-CIMP, ATRX, and IDH mutations have relatively low risk scores, which is also in line with the consensus of scholars. We also explored the correlation between the risk scores and corresponding clinical parameters. We observed that the risk scores were significantly different between patients classified by ATRX status, G-CIMP status, molecular subtype, and IDH1 status (Figures 6E–H). Moreover, we found a significant negative correlation between mRNAsi and risk scores, which is consistent with the opposite survival trend for both (Supplementary Figure S4B). To further measure the performance of the prognostic model, ROC curves were calculated. The ROC curves showed that the predictive model could accurately predict the 3-year survival rates (AUC = 0.871), IDH status (AUC = 0.857), G-CIMP status (AUC = 0.879), and ATRX status (AUC = 0.778) (Figures 6I–L). For each subtype of GBM patients, the statistically significant OS difference between patients with high and low-risk scores was restricted to mesenchymal and proneural GBM patients (Figures 6M–O). These results confirmed that the risk scores derived from the predictive model could accurately predict GBM outcomes and clinical parameters, especially for G-CIMP status.

By univariate Cox analysis, age and risk scores were risk factors with hazard ratios (HRs) > 1, while mRNAsi and IDH status were protective factors with HRs < 1. A similar trend of risk scores was also observed when including these factors in the multivariate Cox proportional hazards regression (Figures 6P,Q). These results indicated that the risk scores could predict the prognosis of GBM patients independently.

### Verification of Prognostic Model Capabilities

To further determine whether these findings from the TCGA database were also applicable to other GBM cohorts, we downloaded and analyzed the gene expression data of 388 GBM patients from the CGGA database. 14 genes were selected for subsequent analysis by univariate analyses (*P* < 0.3). Different from the above predictive model, the optimal value for this lambda was 3 (Partial Likelihood Deviance takes the minimum value), which means that three of the 14 hub genes are selected as candidate genes for the subsequent model construction. Next, the same LASSO algorithm based on the optimal lambda value was applied, and significant differences in OS were observed between patients stratified by the median risk score (Supplementary Figures S5A–C).

The heatmap showed the expression of the three selected genes in high- and low-risk patients in the CGGA dataset. We observed significant differences between the high- and low-risk groups with respect to IDH status (*P* < 0.001) and 1p/19q codeletion status (*P* < 0.001) (Supplementary Figure S5D). Of course, we also measured the relationship between risk scores and clinical traits. We observed that the risk scores were significantly different between patients stratified by IDH status and 1p/19q codeletion status (Supplementary Figures S5E,H). We also observed that patients with 1p/19q codeletion and IDH mutations possessed lower risk scores, which is also a testament to the fact that patients with 1p/19q codeletion or IDH mutations have a better prognosis. The ROC curve also showed high performance in predicting the 1p/19q codeletion status and 3-year survival rate (Supplementary Figures S5F,G). These results indicate that the risk scores could accurately predict the outcomes of patients with GBM and their clinical features, especially the 1p/19q codeletion status. Besides, by univariate and multivariate Cox analyses, sample type and risk scores were selected as risk factors with HRs > 1 (*P* < 0.05), which indicates that the risk scores could independently predict the prognosis of GBM patients (Supplementary Figures S5I,J). Based on the good performance in the construction and verification process, our prediction model is a promising biomarker that can be used to evaluate the prognosis and molecular parameters of GBM.

### Validation of the Hub Genes in Clinical Samples

**FIGURE 7:**
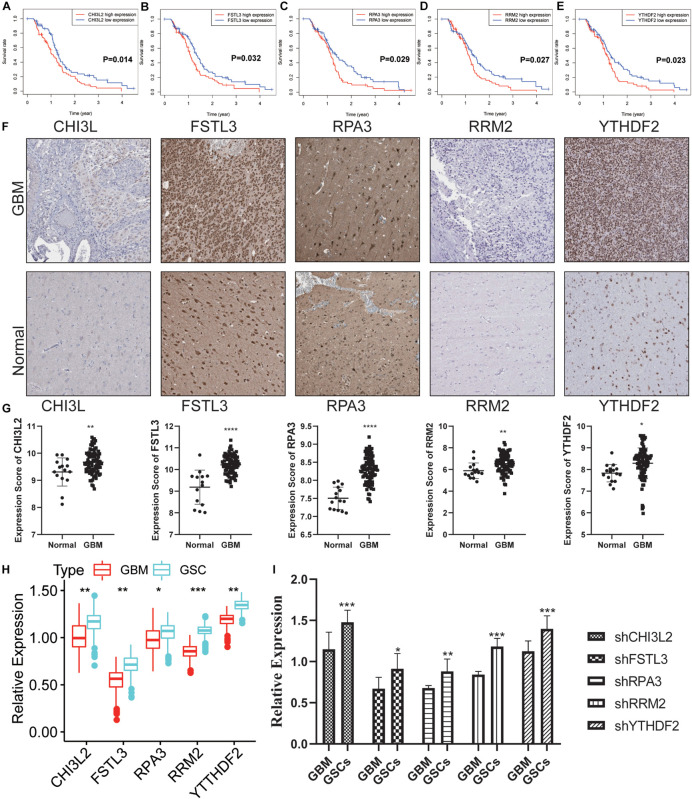
Validation of the DEGs in clinical samples. **(A–E)** Kaplan-Meier survival curves for patients of GBM with high and low gene expression in the TCGA dataset. **(F)** The protein expression of CHI3L2, FSTL3, RPA3, RRM2, and YTHDF2 in clinical human GBM tissue and normal tissue was detected by IHC. Whole tissue photos are shown (100× and 400×). **(G)** Protein expression scores in GBM tissues and normal brain tissues. ***P < 0.001. **(H)** The five hub genes were verified in the GEO database. In GSE124145, the gene expression value of hub genes was higher in glioblastoma stem cells (GSCs) (three samples) than that in GBM (three samples). **(I)** Relative expression of these five hub genes in GSCs and GBM cells. ****P < 0.0001, ***P < 0.001, **P < 0.01, *P < 0.05, ns, no significance.

To explore the potential role of the individual hub gene in OS, we generated the K-M survival curve from the TCGA database. 5 of the 18 hub genes showed significant predictions of poor OS (*P* < 0.05, Figures 7A–E). Subsequently, we performed clinical retrospective studies on the expression of these five proteins. As shown in Figures 7F,G, the expression levels of five proteins in GBM tissues were significantly higher than those in normal brain tissues. To explore the expression levels of five hub genes in GBM and GSCs, we searched the GEO database (GSE124145) and found that these hub genes were significantly overexpressed in GSCs (Figure 7H). In addition, we found the same significant trend of elevated expression on NCH64436 (Figure 7I). The above results indicated that these hub proteins encoded by the hub genes may play a feasible oncogenic role in the stemness of GBM.

### Knockdown of the Hub Proteins

**FIGURE 8:**
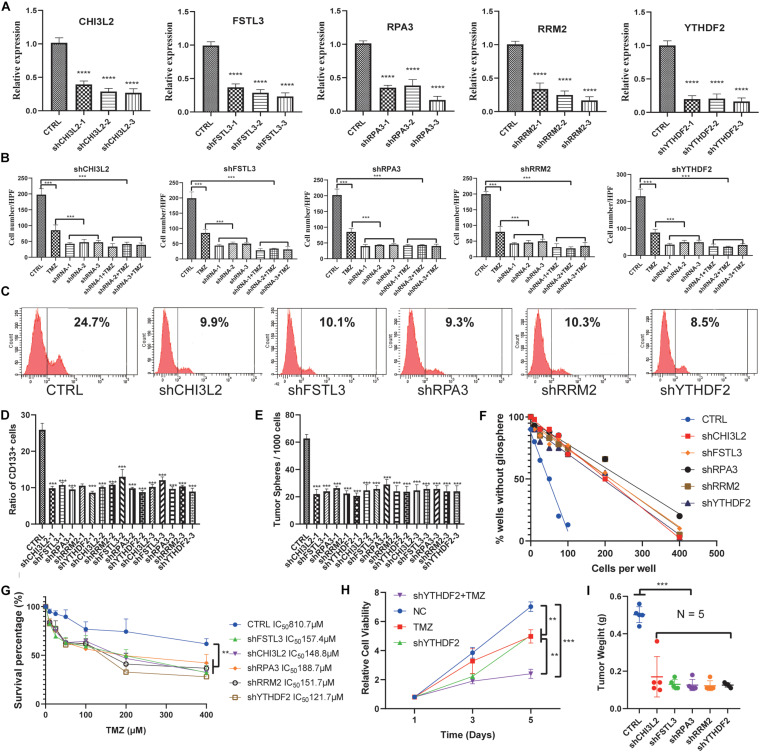
Knockdown of the hub proteins. **(A)** The expression level of the hub genes in glioblastoma stem cells transfected with the corresponding shRNA. **(B)** Cells transfected with the corresponding shRNA that had passed through the membrane were counted. **(C)** Representative figures of the proportion of flow cytometric analysis of CD133+ cells. **(D)** Quantification of the proportion of CD133+ cells from different shRNA-treated stem cell lines. **(E)** Evaluation of the number of spheres from 1000 cells. The number of primary spheres formed on day 9 is shown. **(F)** Tumor sphere formation was measured through a limiting dilution assay (*n* = 48 wells/condition, *P* < 0.05). **(G)** The survival percentage was determined by the MTT assay at 490 nm. NCH64436 cells stably expressing CTRL or shRNA were exposed to TMZ over the range of 10–400 μM for 48 h followed by cell viability analysis. **(H)** MTT assays of the effects of TMZ on NCH64436 cells at different time points. NCH64436 cells with control or shYTHDF2 were treated with 200 μM of TMZ for the indicated time, cell viability was analyzed by MTT assays. **(I)** The weights of control (*n* = 5) and each knockdown (*n* = 5) tumors. ** *P* < 0.01, ***P < 0.001, ****P < 0.0001. All data are represented by mean ± SEM.

To further explore the potential role of these hub proteins in GBM stem cells, we performed MTT, transwell, and flow cytometric assay. Human GSCs (NCH64436) were transiently transfected with shRNAs, and the efficiency of transfection was confirmed by qRT-PCR (Figure 8A). It is well known that GSC is extremely resistant to TMZ. Excitingly, the combination of shRNAs and TMZ works synergistically (Figure 8B and Supplementary Figure S6A). These results confirmed that the migration ability of GSC was significantly reduced after the hub gene was knocked down. As a key molecule to maintain the stemness of tumor cells, CD133 is a common surface marker in stem cells or progenitor cells, especially in the nervous system (Wilson et al., 2004; Rusu et al., 2019). We examined the ratio of CD133 in stem cells with different shRNAs by flow cytometry. As shown in Figures 8C,D, the ratio of CD133+ cells with hub genes knocked-down was significantly reduced. These results suggested that the hub gene could serve as a key molecule in regulating GSCs expression of CD133.

Next, we conducted a limiting dilution assay to analyze tumorsphere formation at a continuous cell concentration of 400 to 5 cells/well in the GSC cell line. Consistently, all the isolated GSC showed the self-renewal capacity to develop tumorspheres. However, GSC in which the hub genes were knocked down generated a small number of tumorspheres (Figure 8E). Moreover, the number of cells required to generate at least 1 tumorsphere/well was determined to be 127.90 in control, and >400 in hub genes knocked down (Figure 8F and Supplementary Figures S6B,C).

MTT was applied to test the viability of GSCs. It is worth noting that YTHDF2, as a reader of RNA m6A methylation, has been validated to be related to TMZ resistance in GSCs (Du et al., 2020). We found that TMZ and shYTHDF2 could synergistically inhibit the viability of GSCs (Figure 8H). Besides, the IC50 of TMZ-treated GSCs was 810.7 μM, while the IC50 of GSCs with the hub gene knocked down was significantly decreased. Among them, the IC50 of shYTHDF2 was the smallest, which was 121.7 μM (Figure 8G). We also found that two other sets of shRNAs showed the same MTT trend, eliminating the possibility of an off-target effect induced by gene knockdown (Supplementary Figures S6D,E).

To study the capacity of hub genes to inhibit tumor initiation *in vivo*, we established a xenograft model using GSCs. We found that knockdown of the hub gene significantly inhibited tumor growth. Tumor weight was significantly reduced in the knockdown group compared to the control group (Figure 8I, Supplementary Figure S6F, and Supplementary Table S4). These results indicated that knockdown of the hub genes reduced the proliferation and migration of GSCs.

## Discussion

Using a large cohort of GBM transcriptome data based on the combination of clinical information and gene expression profiles, we conducted an integrated analysis of GBM stemness and determined their prognostic and diagnostic values. We searched the TCGA data for mRNA expression and clinical parameters. By using two different molecular metrics of stemness derived from a machine learning algorithm, we identified the epigenomic and transcriptomic stemness characteristics of GBM based on clinical/molecular features. By using the ESTIMATE and ssGSEA algorithm, we obtained insight into the association of the stemness of GBM and the immune microenvironment. Moreover, these analyses enabled us to identify potential operational targets (or their MoAs) to pave the way for differentiation therapy for tumors and metastases. By combining WGCNA with differential analysis, we identified 18 stemness-related hub genes, 10 of which were significantly related to survival. Moreover, most hub genes have significant changes (up-regulate or down-regulate) after chemotherapy or radiotherapy. Besides, we derived a prognostic model from 18 stemness-related genes that effectively stratified the OS of patients with GBM into high- and low-risk subgroups. Notably, the performance of this prognostic model was also recognized in the validation set (CGGA, an independent glioma database). Ultimately, survival analysis and *in vitro* experiments confirmed five potential molecular markers that could be considered as potential targets for inhibiting the proliferation of GSCs. The results based on an *in vivo* xenograft model are consistent with the finding that knockdown of the hub gene inhibits the growth of GSCs *in vitro*. Our approach could be applied to facilitate the development of objective diagnostic and targeted treatment tools to quantify cancer stemness in clinical tumors, and perhaps lead considerable benefits that predict tumor prognosis, identify new stemness-related targets and targeted therapies, or improve targeted therapy sensitivity.

At present, surgical resection, radiotherapy, chemotherapy, and other treatment methods have been quite advanced, which have significantly improved the survival of GBM patients. However, due to the presence of peritumoral edema caused by large-scale invasiveness of tumors, recurrence of GBM is inevitable. Survivors hence have to suffer severe neurological side effects, including recurrence of malignant tumors and psychological fear of death (Athanassiou et al., 2005; Monteiro et al., 2017; Lu et al., 2018). The lack of effective treatments for GBM may be due to the invasiveness of the tumors and the therapeutic resistance of GSCs (Singh et al., 2004; Bao et al., 2006; Cui et al., 2017). In the past few years, TCGA has clarified the status of nearly 12,000 patients of 33 cancer types by generating comprehensive data, such as expression profiles, transcriptomes, proteomics, as well as clinicopathological parameters. Such cancer stemness indices associated with cancer dedifferentiation have been completely identified by artificial intelligence and deep machine learning due to the existence of these resources (Malta et al., 2018). Therefore, it is necessary to make breakthroughs in terms of GSCs in GBM.

Among the GBM genetic aberrations, the IDH1/2 mutation (Yan et al., 2009; Turcan et al., 2012) have shown reliable prognostic and/or predictive value compared to their counterparts. The exploration of the GBM with G-CIMP was another milestone during the process of exploring epigenetics (Noushmehr et al., 2010). Patients harboring G-CIMP (G-CIMP+) tumors that are strictly related to the IDH1/2 mutation have shown a significantly delayed mortality that was not observed in their counterparts (Brennan et al., 2013). MRNAsi was highest in normal samples, decreased in primary tumors, and lowest in recurrent, which is consistent with the conception that oncogenic dedifferentiation is generally involved in tumorigenesis and development. In fact, most of the mRNAsi of 33 cancers in TCGA are negatively correlated with survival. The positive trend of correlation in GBM mRNAsi may be partly explained by the dominant genomic alteration associated with the GBM tumor type. Roughly 80% of GBM tumors carry an IDH1/2 mutation and, as demonstrated by our group and others, confer a G-CIMP (Turcan et al., 2012). The relationship between EMT and stemness has always been a hot topic. A set of studies showed that EMT was necessarily related to stemness (Fabregat et al., 2016). Interestingly, positive associations were observed between EMT critical proteins and stemness. This is because most of the TCGA data come from primary tumors in the pre-EMT status. These tumors are basically epithelial cells despite the degree of dedifferentiation. However, EMT is closely related to tumor recurrence and metastasis. Mesenchymal characteristics could be acquired by accumulating additional mutations or epigenetic changes in the tumor. These cells with mesenchymal characteristics can be spread to other organs through blood, lymph, and internal transmission pathways to regain the epithelial phenotype, as well as form metastatic or recurrent tumors. PD-L1 on cancer cells can prevent the activation and recruitment of immune cells in lymph nodes by binding to the PD1 receptor on T cells, thus helping cancer cells escape the surveillance of the immune system (Chen and Mellman, 2013). The negative correlation between PD-L1 expression and stemness indicates that GBM is not susceptible to treatment with targeted immune therapies. Also, mRNAsi was significantly associated with all clinical features, including OS. From these results, one may conclude that GSCs are related to tumorigenesis, tumor recurrence, tumor prognosis, and molecular characteristics. Further analysis of the stemness indices may update our understanding of the value of GSCs.

Tumors grow in complex, diverse, and complete ecosystems of cancer stem cells, relatively differentiated cancer cells, tumor-associated stromal cells, infiltrating immune cells, and other cell types. This ecosystem, defined as a “hotbed” of tumor formation, is frequently characterized by hypoxia and abnormal levels of inflammatory factors, various cytokines, and immune components (Lyssiotis and Kimmelman, 2017). We found that mRNAsi had a negative association with the immune/stromal scores, suggesting that stem cells have the propensity to promote the loss of immune cells. A growing number of studies have shown that the inoculation of embryonic stem cells or induced pluripotent stem cells can increase the specific immune response to cancer cells, thereby emphasizing the common features between cancer cells and stem cells during the immune response (Kooreman et al., 2018). The progression of cancer is inversely related to the host’s immunocompetence, and there is evidence that CSCs play a modulatory role in the immune system (Kawasaki and Farrar, 2008; Schatton and Frank, 2009). GSCs reside in an environment that protects them from immune system attacks. Residential zones could attract immunosuppressive cells such as M2 macrophages and regulatory T cells. We speculate that the stemness index may help predict the efficacy of stem cell-associated immunotherapy and help determine which patients will respond to such treatments.

Glioblastoma multiforme stem cells are the major mechanism of GBM resistance to radiotherapy and chemotherapy, so finding genes related to stemness is an important step to solve the resistance problem. 18 hub genes were identified and validated. However, not all, but most hub genes have significantly different expression levels after chemoradiotherapy. Traditional DNA repair systems also contribute to chemotherapy resistance. The yellow module, which was positively correlated with mRNAsi, was related to the development of stem cell differentiation and dedifferentiation characteristics. The functional annotation of this module was primarily associated with stem cell self-renewal and proliferation. Through Oncomine analysis, all hub genes were highly expressed in different cancers. Besides, the overexpression of some hub genes was related to the level of stemness, and their continuous expression changes might promote tumor progression and post-treatment progression. Most of these genes have been reported in GBM to be related to the characteristics of GSCs. Aurora kinase A (AURKA), as an ATM kinase, was reported to cause radio-resistance through self-activation (De Bacco et al., 2016). Xenopus kinesin-like protein 2 (TPX2) was reported to promote tumor invasion and proliferation (Chockalingam and Ghosh, 2013). Notably, through protein interaction and co-expression networks, we speculate that AURKA, CCNA2, and TPX2 are ideal drug targets in GBM, which provide potential evidence for stem cell treatment in GBM. First, these two targets were significantly overexpressed in GBM. Second, they were screened out from the most significant module and formed a network with closely connected interactions. Finally, their correlation was quite strong (*P* < 0.05).

Connectivity Map can identify biomarkers for predicting specific drug reactions, mechanisms of treatment, and ways to overcome resistance (Petrich et al., 2012; Leshchenko et al., 2014; Xiong et al., 2019). We queried CMap using the DEGs of the mRNAsi grouping. CMap analysis accurately identified numerous compounds that have been shown to have an effect on CSCs of other tumor types with specificity (Subramanian et al., 2017; Brum et al., 2018; Malta et al., 2018; Lian H. et al., 2019). HDAC inhibitors have been reported to be potent differentiation agents in GSCs, reducing GBM growth mainly by inducing cell necrosis and growth arrest (Tung et al., 2018). Also, a cell cycle inhibitor (Tachon et al., 2018), a dopamine receptor antagonist (Dolma et al., 2016), an adrenergic receptor antagonist (He et al., 2017), a VEGFR inhibitor (Kalpathy-Cramer et al., 2017), a PPAR receptor agonist (Im, 2016; Gupta et al., 2018), a PI3K inhibitor (Zhao et al., 2017), mTOR inhibitors (Friedman et al., 2013), a lipoxygenase inhibitor (Zappavigna et al., 2016), and an HMGCR inhibitor (Hamm et al., 2014) have been shown to exhibit anticancer effects on GBM cells, some of which have been shown to target GSCs. Our findings are expected to facilitate the development of antitumor strategies that specifically target GSCs and pave the way for the treatment of cancer resistance.

Cancer stem cell models derived from 16 acute myeloid leukemia (AML) samples demonstrated an extraordinary ability to predict the clinical outcome of AML (Eppert et al., 2011). Similarly, in breast cancer, colon cancer, gastric cancer, and non-small cell lung cancer, the stem cell gene profile model holds promise in redefining clinical outcome stratification and identifying reduced risk (Giampieri et al., 2013; Miao et al., 2017; Codony-Servat et al., 2019; Neckmann et al., 2019). Moreover, three miRNAs (miR-23a, miR-9-3p, and miR-27a) derived from the analysis of GSCs by gene expression profiling, grouped mesenchymal and proneural GBM patients into two different categories with significant survival differences (Marziali et al., 2017). These studies reveal that cancer stem cell markers showed relatively high performance in predicting clinical outcomes for a variety of tumors, including GBM. In this current study, we established and validated an 18-mRNA-based prognostic model related to the stemness. To the best of our knowledge, this prognostic model has not been reported for GBM and may potentially provide guidance for developing novel clinical management strategies. One of the significant advantages of the predictive model is that it does not require the identification of somatic mutations and molecular subtypes in patients, which may make the detection based on mRNA expression profiles more routine. Additionally, concerning single-cell transcriptome sequencing in GBM patients, the model could indicate patterns of molecular heterogeneity within the tumor and identify the degree of oncogenic dedifferentiation.

The intra-tumor heterogeneity of GBM suggests that GSCs existing in the tumor microenvironment are effective in stimulating residual tumor progression or recurrence (Wilson et al., 2004). Our survival analysis and *in vitro* study showed that CHI3L2, FSTL3, RPA3, RRM2, and YTHDF2 were beneficial to the proliferation, invasion, and chemoresistance of GSCs. Chitinase 3-like-2 (CHI3L2, also known as CHI3l3), a famous biomarker for selective activation of macrophages and microglia, has been reported to activate epidermal growth factor receptor (EGFR), which determines the fate of neural stem cells transformed into oligodendrocyte lines via Chi3l3-EGFR-Pyk2 signaling axis (Starossom et al., 2019). This is consistent with our finding that CHI3L2 regulates the expression of the stemness marker CD133 in GSC. We further confirmed the importance of knocking down CHI3L2 and chemotherapy resistance could synergistically inhibit the proliferation of GSC, which paved the way for the next stem cell-targeted therapy strategy. FSTL3 has been reported to promote the transformation of pluripotent stem cells to endothelial, cardiogenic (Genovese et al., 2009; Kelaini et al., 2018). FSTL-3 has also been reported to be independently associated with malignant progression of breast cancer and tumor size (Panagiotou et al., 2019). However, its role in tumor stem cells remains unclear. As far as we know, this is the first study on the role of FSTL3 in tumor stem cells. We predict that FSTL3 may have similar functions in other tumors because of its independent prognostic role in tumors. RPA3, a member of the replication protein family, plays an important role in DNA repair, recombination, replication, and cell cycle regulation. RPA3 is associated with the occurrence and poor prognosis of liver cancer, as well as the aging of hematopoietic stem cells (Xiao et al., 2018; Lian X. et al., 2019; Zhang and Yu, 2020). RRM2 has been reported to be associated with the occurrence and poor survival of prostate cancer. The main effect mechanism is the overexpression of DNA damage repair genes, which is related to stem cell differentiation (Mazzu et al., 2019; Wei et al., 2020). These findings are consistent with our findings, demonstrating that RRM2 may control the stemness of cancer cells by repairing DNA. Unlike the above four genes, YTHDF2 has been determined to be unnecessary for normal functions in a variety of stem cells. However, it can function as a cancer-driving gene to prevent stem cell differentiation and gain self-renewal (Paris et al., 2019). The regulatory effect of YTHDF2 on stem cells on cancer has been confirmed in many cancers, such as acute leukemia and osteosarcoma. However, its effect on GSCs has not been confirmed. Our knockdown of YTHDF2 is to a certain extent consistent with the predecessors’ views, and also confirmed the important role of YTHDF2 on GSC stemness. The molecular and functional characterization of these identified genes may be useful for the development of novel cancer-targeted drugs and the recognition of GSCs.

There are still other restrictions in our analysis. The employment of DEGs in WGCNA may artificially exclude other potential stemness-related genes. Besides, the five normal samples provided by TCGA are a little unbalanced compared to 169 tumor samples. The knockout animal model should also be applied to further explore the effect of hub genes targeting the stemness of GSCs.

## Conclusion

In summary, our study provided a stemness-related prognosis and diagnostic value of GBM by systematically analyzing the stemness characteristics. Our analysis of the interaction between immune cells and GBM stemness might be helpful in predicting the effectiveness of immunotherapy against GSCs and might to determine patients who are sensitive to such therapies. The mRNAsi-based prognostic model might contribute to the individualized prediction of GBM prognosis and serve as a possible biomarker reflecting GBM patients’ response to chemoradiotherapy. The stemness-related targets we identified provide guidance for a synergistic therapeutic strategy for glioma. Our study also provided strategies for the comprehensive analysis of cancer genomics based on machine learning methods for the systematic identification of specific stem cell-related targets and specific targeted drugs based on GBM stemness. However, the conclusions are derived from retrospective data, hence, future investigations are expected to focus on functionally interpreting and validating our findings.

## Data Availability Statement

All datasets presented in this study are included in the article/Supplementary Material.

## Ethics Statement

The animal study was reviewed and approved by Ethics Committee of Harbin Medical University (SYDW-2019-8-2). Written informed consent was obtained from the owners for the participation of their animals in this study.

## Author Contributions

SH, JD, RX, and LC conceived and designed the study and drafted the manuscript. JD and KH provided analytical technical support. XY, YL, HJ, SMi, YB, PZ, and SMa participated in the production of charts and pictures. All authors have read and approved the final manuscript.

## Conflict of Interest

The authors declare that the research was conducted in the absence of any commercial or financial relationships that could be construed as a potential conflict of interest.

## Footnotes

1 https://portal.gdc.cancer.gov

2 www.cgga.org.cn

3 http://www.perl.org/

4 http://www.oncomine.org

5 https://www.string-db.org

6 https://clue.io/
