# Supplementary material for: Identification of Prognostic Model and Biomarkers for Cancer Stem Cell Characteristics in Glioblastoma by Network Analysis of Multi-Omics Data and Stemness Indices
Source: Front Cell Dev Biol. 2020 Oct 19;8:558961. doi: 10.3389/fcell.2020.558961 (PMC7604309; doi:10.3389/fcell.2020.558961)
Supplement: Supplementary file 1 [file Table_1.DOCX]

## Supplementary Figure

**Supplementary Fig. S1**


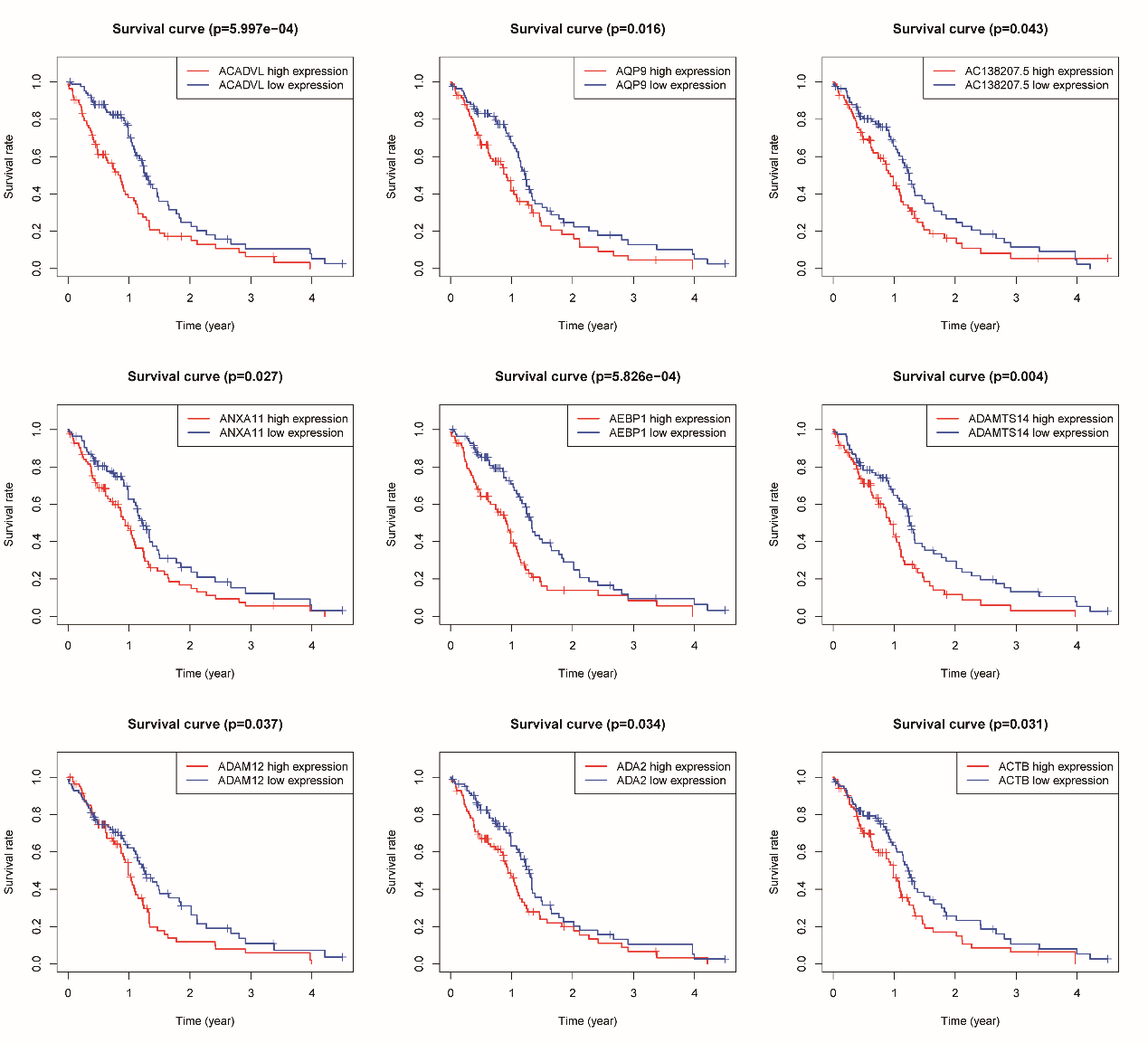


**Supplementary Fig. S1. Representative figures of 181 genes significantly related to poor prognosis.**

**Supplementary Fig. S2**


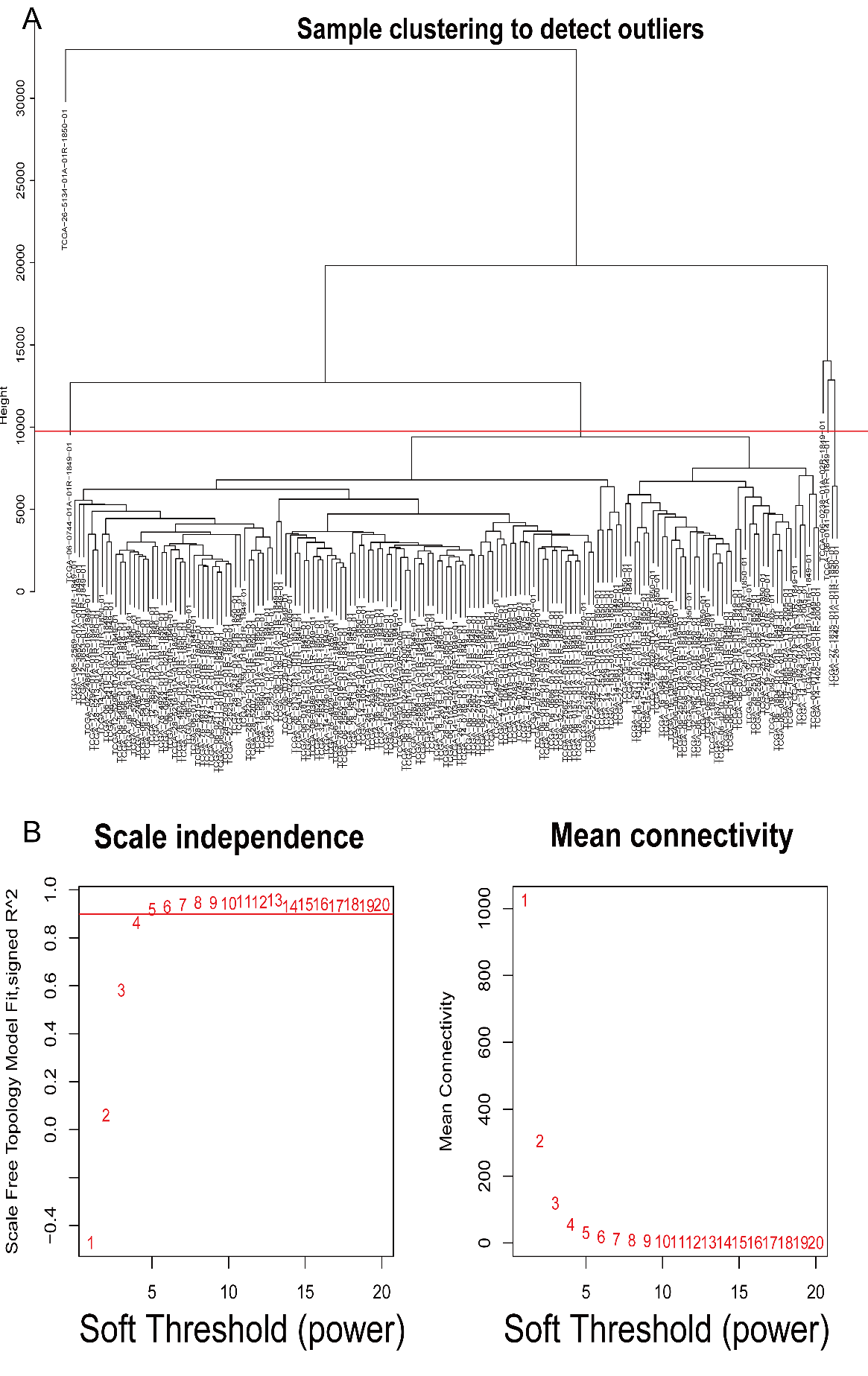


**Supplementary Figure S2. The process of WGCNA.**

**(A)** Clustering of samples and removal of outliers. **(B)** Analysis of network topology for various soft-thresholding powers in scale independence and mean connectivity.

**Supplementary Fig. S3**


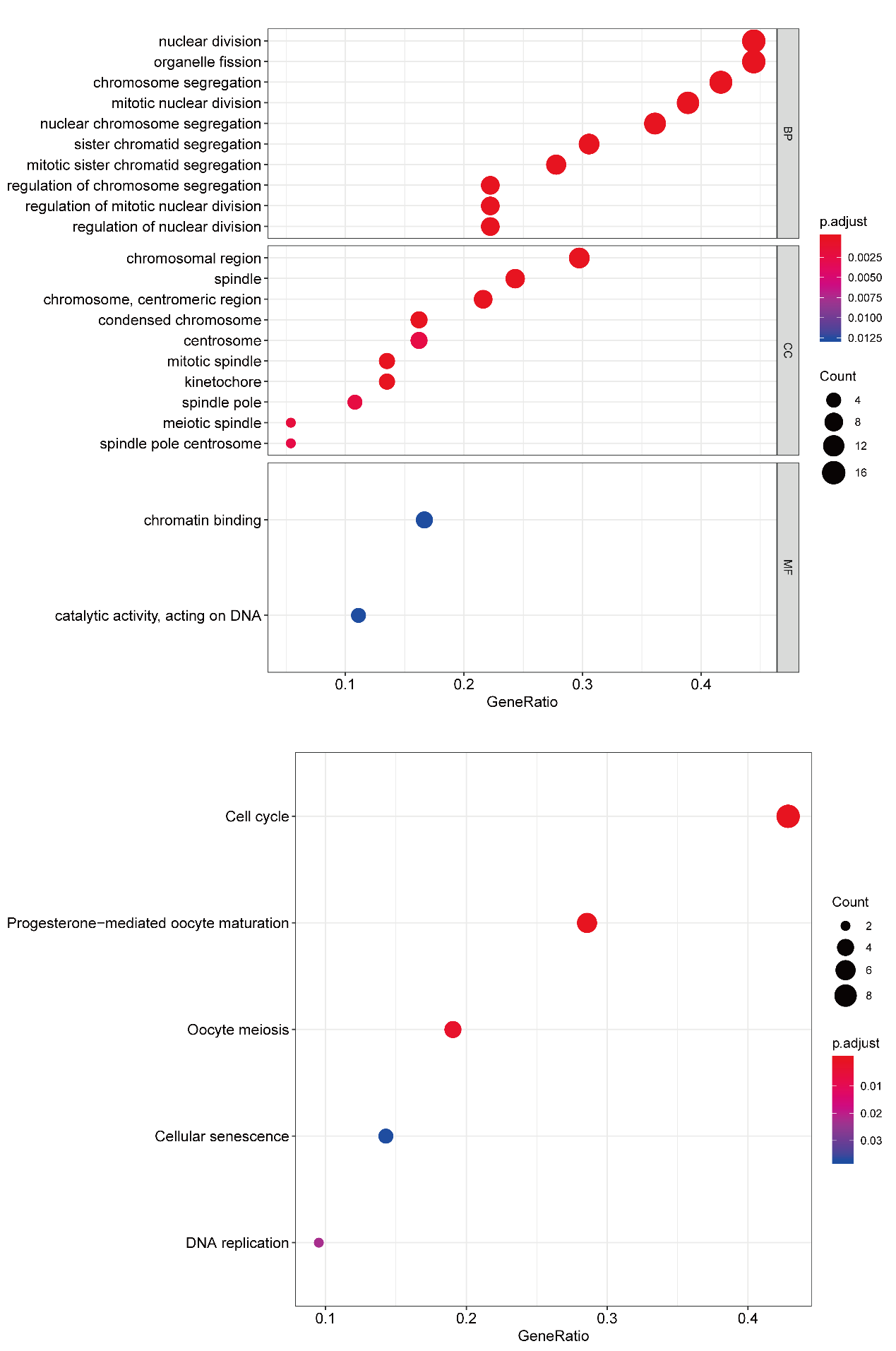


**Supplementary Figure S3. GO and KEGG enrichment analyses of modules and key genes of interest. Under a threshold of P < 0.01 and FDR < 0.05, the top 10 enriched categories of biological process (BP), cellular component (CC), molecular function (MF), and KEGG pathways are listed.**

**Supplementary Fig. S4**


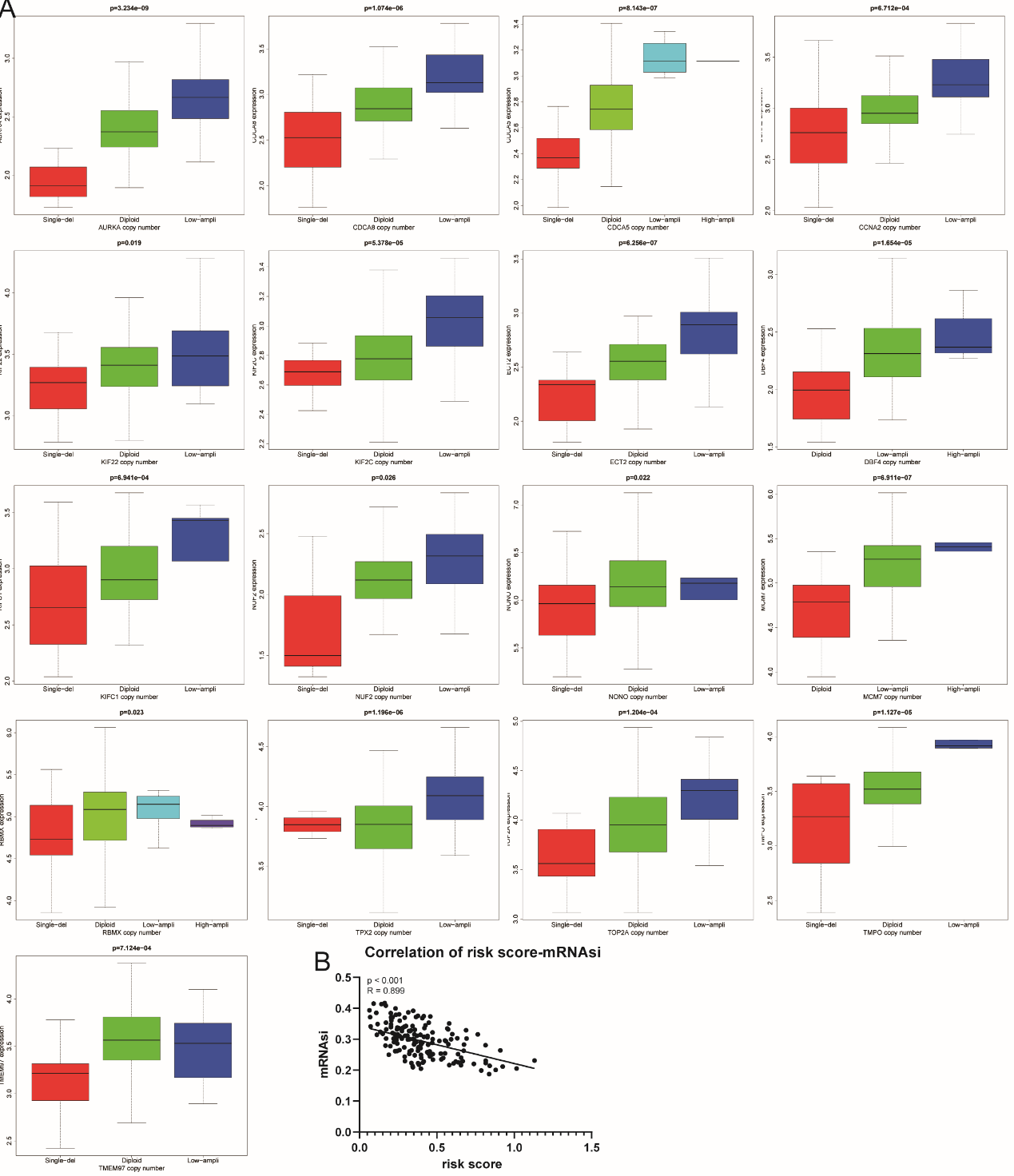


**Supplementary Fig. S4. Subsequent analysis of the hub gene.**

(**A**) The relationship between the copy number variation state of the hub gene and the hub gene expression value. (**B**) The correlation between mRNAsi and risk scores.

**Supplementary Fig. S5**


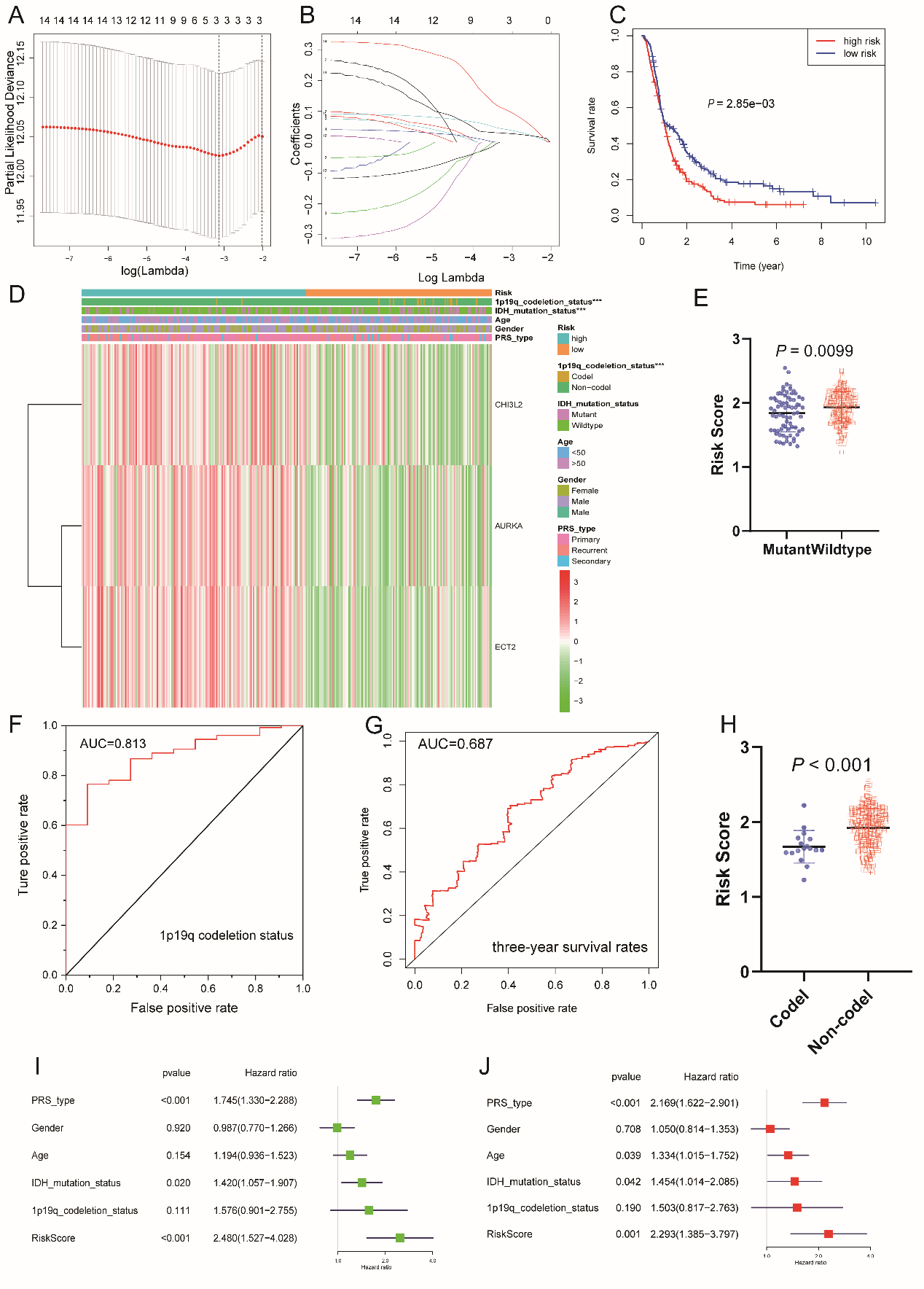


**Supplementary Fig. S5. Construction and confirmatory analysis of the prognostic model based on the hub genes from the CGGA dataset.**

**(A)** Ten-time cross-validation for tuning parameter selection in the LASSO model. **(B)** LASSO coefficient profiles of 13 prognostic genes. **(C)** K-M curves for patients in the CGGA dataset assigned to high- and low-risk groups based on the risk score. **(D)** The heatmap shows the expression levels of the three hub genes in low- and high-risk GBM patients from the CGGA dataset. The distribution of clinicopathological features was compared between the low- and high-risk groups. **(E)** Distribution of risk scores in the CGGA dataset stratified by ATRX status. (F-G) ROC curves showed the predictive efficiency of the predictive model for the 1p/19q codeletion status **(F)** and a three-year survival rate **(G)**. **(H)** Distribution of risk scores in the CGGA dataset stratified by 1p/19q codeletion status. **(I-J)** Univariate **(I)** and multivariate **(J)** Cox regression analyses of the association between the clinicopathological factors (including the risk score) and overall survival of patients in the CGGA dataset. ****P* < 0.001, ***P* < 0.01, * *P* < 0.05.

**Supplementary Fig. S6**


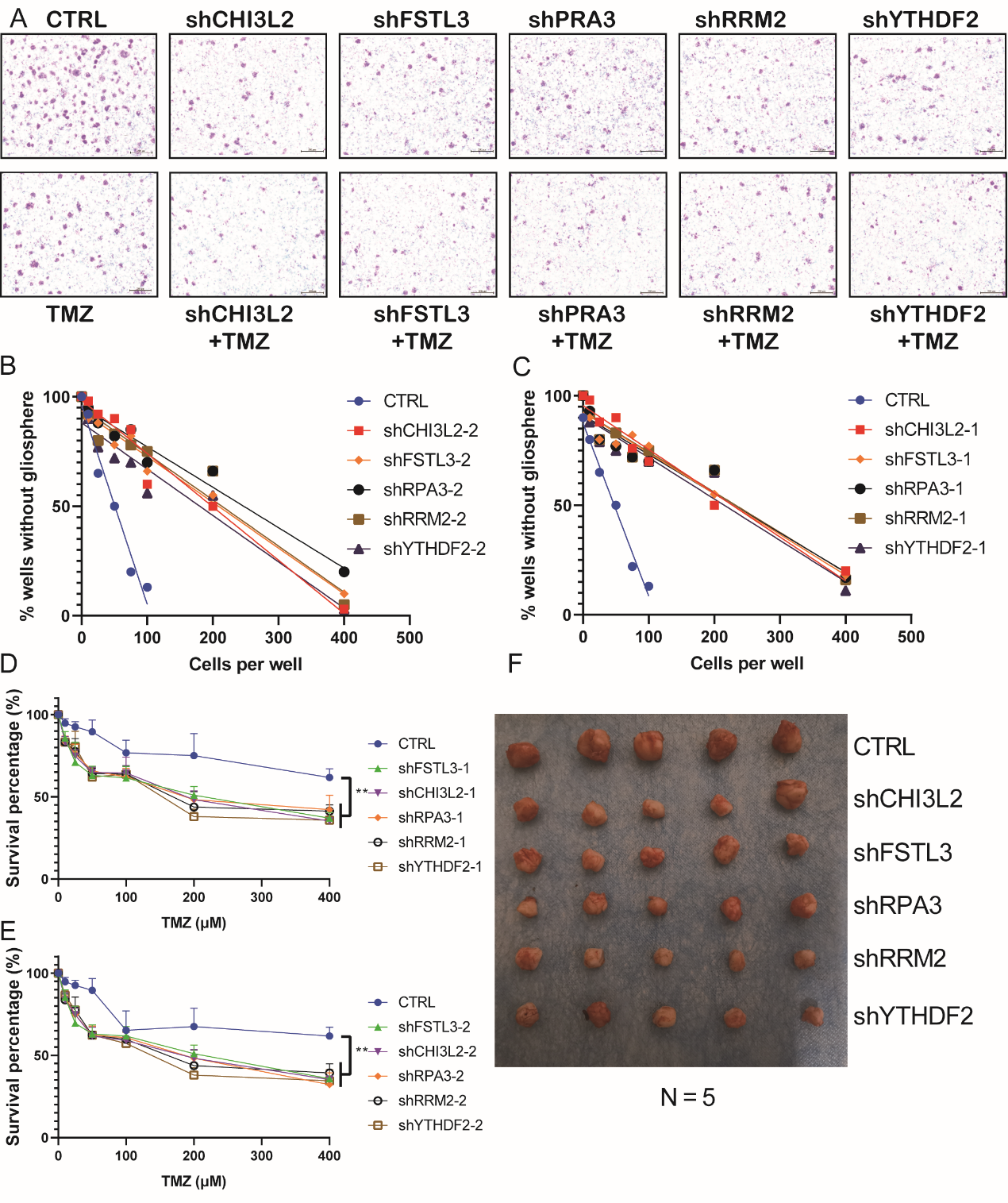


**Supplementary Fig. S6. Sphere-forming and limiting dilution assays.**

**(A)** Transwell assay in specific shRNA transfected or control cells. Representative images are shown. **(B-C)** Tumor sphere formation was measured through a limiting dilution assay (n=48 wells/condition, *P* < 0.05). **(D-E)** The survival percentage was determined by MTT assays at 490 nm. NCH64436 cells stably expressing CTRL or shRNA were exposed to TMZ over the range of 10-400 μM for 48 h followed by cell viability analysis. * *P* < 0.05 ** *P* < 0.01, *** *P* < 0.001. All data are represented by mean ± SEM. (**F**) Cells with control and knockdown hub genes were injected into the nude mice. Tumors derived from cells were removed from the mice (n = 5). * *P* < 0.05 ** *P* < 0.01, *** *P* < 0.001. All data are represented by mean ± SEM.
