# Supplementary material for: Identification of Prognostic Model and Biomarkers for Cancer Stem Cell Characteristics in Glioblastoma by Network Analysis of Multi-Omics Data and Stemness Indices
Source: Front Cell Dev Biol. 2020 Oct 19;8:558961. doi: 10.3389/fcell.2020.558961 (PMC7604309; doi:10.3389/fcell.2020.558961)
Supplement: Supplementary file 2 [file Table_2.DOCX]

## Table S1. Clinicopathological features of patients included in this study.

|  |  | TCGA dataset | | CGGA dataset | |
| --- | --- | --- | --- | --- | --- |
|  |  | Number | Percentage | Number | Percentage |
| Total |  | 174 | 100% | 388 | 100% |
| N/T |  | | | | |
|  | Normal | 5 | 2.87% | 0 |  |
|  | Tumor | 169 | 97.12% | 388 | 100% |
| Gender |  | | | | |
|  | Female | 59 | 34.91% | 153 | 39.43% |
|  | Male | 110 | 65.08% | 235 | 30.57% |
| Subtype |  | | | | |
|  | Classical | 42 | 25.45% | NA |  |
|  | Mesenchymal | 56 | 33.94% | NA |  |
|  | Neural | 28 | 16.97% | NA |  |
|  | Proneural | 39 | 23.64% | NA |  |
| P/R |  | | | | |
|  | Primary | 156 | 92.31% | 225 | 58.00% |
|  | Recurrence | 13 | 7.69% | 163 | 42.00% |
| Fustat |  | | | | |
|  | Dead | 118 | 69.82% | 289 | 81.875 |
|  | Alive | 51 | 30.18% | 64 | 18.13% |
| Age |  | | | | |
|  | <= 65 | 109 | 64.50 % | 29 | 7.47% |
|  | > 65 | 60 | 35.50 % | 359 | 92.53% |
| G-CIMP status |  |  |  |  |  |
|  | G-CIMP | 11 | 6.67% | NA |  |
|  | Non-G-CIMP | 154 | 93.32% | NA |  |
| IDH status |  | | | | |
|  | Mutant | 9 | 6.0% | 90 | 23.80% |
|  | Wildtype | 140 | 94.0% | 288 | 76.20% |
| 1p19q |  | | | | |
|  | Codel | NA | NA | 20 | 5.65% |
|  | Non-Codel | NA | NA | 334 | 94.35% |

Abbreviations: IDH, isocitrate dehydrogenase; G-CIMP, cytosine-phosphate-guanine island methylator phenotype; Codel, codeletion; NA, Not Applicable.

## Table S2. Primers and target sequences for shRNAs for identified genes.

**Target sequences**

FSTL3

CCGGCCCGGACCTGAGCGTCATGTACTCGAGTACATGACGCTCAGGTCCGGGTTTTTG

CCGGCGGCAACAACAACGTCACCTACTCGAGTAGGTGACGTTGTTGTTGCCGTTTTTG

CCGGTGCCTTCCCTGCAAAGATTCGCTCGAGCGAATCTTTGCAGGGAAGGCATTTTTG

CHI3L2

CCGGGAGACCAAGGTTCAGTTCTTACTCGAGTAAGAACTGAACCTTGGTCTCTTTTTG

CCGGGCTCCTACTACAATGTGGAATCTCGAGATTCCACATTGTAGTAGGAGCTTTTTG

CCGGCAGACCATCAACAGTCTCAAACTCGAGTTTGAGACTGTTGATGGTCTGTTTTTG

RPA3

CCGGGTACATCTTATGTCCAGTTTACTCGAGTAAACTGGACATAAGATGTACTTTTTG

CCGGTCTGGAATTGTGGAAGTGGTTCTCGAGAACCACTTCCACAATTCCAGATTTTT

CCGGGCTAGCTCAATTCATCGACAACTCGAGTTGTCGATGAATTGAGCTAGCTTTTT

RRM2

CCGGGCAGACAGACTTATGCTGGAACTCGAGTTCCAGCATAAGTCTGTCTGCTTTTTG

CCGGCCCATTTGACTTTATGGAGAACTCGAGTTCTCCATAAAGTCAAATGGGTTTTTG CCGGGCTCAAGAAACGAGGACTGATCTCGAGATCAGTCCTCGTTTCTTGAGCTTTTTG

YTHDF2

CCGGGCTACTCTGAGGACGATATTCCTCGAGGAATATCGTCCTCAGAGTAGCTTTTTG

CCGGCCACAGGCAAGGCCCAATAATCTCGAGATTATTGGGCCTTGCCTGTGGTTTTTG

CCGGCGGTCCATTAATAACTATAACCTCGAGGTTATAGTTATTAATGGACCGTTTTTG

**Primers**

YTHDF2

TCTGTCCACTCCTCTGCTATT

CATTCTGCACCCTGTCCTTATC

RRM2

Forward CGAACGCTCAAGTGAAACAAG (Sense)

Reverse TCTTCCACGGAGACCAGAA (AntiSense)

RPA3

Forward GACTCCACTTGAGCAACTCTAC (Sense)

Reverse TCCACCCTTCACAACGAAC (AntiSense)

CHI3L2

Forward CCTTCCTATGCTCTCATCTCATC (Sense)

Reverse CTGAACCTTGGTCTCCATACTC (AntiSense)

FSTL3

Forward GTCTGCAGAAGAGGAAGAGAAC (Sense)

Reverse TCCTAGAGAAGGGCCCTAAAT (AntiSense)

Table S3. Compounds with an enrichment score ≤ -95 that could target pathways associated with GBM stemness.

| name | | | description |
| --- | --- | --- | --- |
| oligomycin-a | ATP synthase inhibitor, ATPase inhibitor | target | Score |
| fluoropyruvate | PDH inhibitor | ATP5A1 | -99.75 |
| HLI-373 | MDM inhibitor |  | -99.72 |
| n-(3-acetamidophenyl)-3-chlorobenzamide | glutamate receptor inhibitor | MDM2 | -99.44 |
| L-2167 | PPAR receptor agonist | GRM5 | -99.44 |
| valproic-acid | HDAC inhibitor, ABAT inhibitor, GABA receptor agonist, GABAergic transmission enhancer, voltage-gated sodium channel blocker | PPARD | -99.26 |
| deferiprone | chelating agent, cytochrome P450 inhibitor, iron absorption inhibitor, reducing agent | ABAT, HDAC1, SCN1A, SCN3A, ACADSB, ALDH5A1, HDAC2, HDAC9, OGDH, SCN10A, SCN11A, SCN1B, SCN2A, SCN2B, SCN3B, SCN4A, SCN4B, SCN5A, SCN7A, SCN8A, SCN9A | -99.05 |
| BRD-K07872006 | lipoxygenase inhibitor | CYP4F2 | -99.05 |
| nornicotine | acetylcholine receptor agonist |  | -98.94 |
| clofibrate | PPAR receptor agonist |  | -98.7 |
| U-46619 | thromboxane receptor agonist | PPARA, LPL | -98.7 |
| losartan | angiotensin receptor antagonist | PTGDR, PTGDR2, PTGER1, PTGER2, PTGER3, PTGER4, PTGFR, PTGIR, TBXA2R | -98.68 |
| BRD-A05680309 | caspase activator, XIAP inhibitor | AGTR1 | -98.61 |
| amisulpride | dopamine receptor antagonist | CASP8, XIAP | -98.54 |
| PKCbeta-inhibitor | PKC inhibitor | DRD2, DRD3, HTR2A, HTR7 | -98.36 |
| dextromethorphan | glutamate receptor antagonist, sigma receptor agonist | PRKCB | -98.31 |
| sitagliptin | dipeptidyl peptidase inhibitor, HMGCR inhibitor, insulin secretagogue, tumor necrosis factor expression inhibitor | SIGMAR1, CHRNA2, CHRNA3, CHRNA4, CHRNA7, CHRNB2, CHRNB4, CYBA, CYBB, CYP3A5, GRIN1, GRIN3A, NCF1, NCF2, NCF4, OPRD1, OPRK1, OPRM1, PGRMC1, RAC1, RAC2, SLC6A2, SLC6A4 | -98.26 |
| pilocarpine | acetylcholine receptor agonist | DPP4, CYP2C8, FASLG, HMGCR, SLC22A8 | -98.18 |
| maraviroc | CC chemokine receptor antagonist, CC chemokine receptor 5 (CCR5) antagonist, reverse transcriptase inhibitor | CHRM3, CHRM1, CHRM2, CHRM4, CHRM5 | -98.03 |
| tyrphostin-AG-112 | protein tyrosine kinase inhibitor | CCR5, CYP3A5 | -97.96 |
| naftopidil | adrenergic receptor antagonist, calcium channel antagonist | EGFR | -97.73 |
| 3-amino-benzamide | PARP inhibitor | ADRA1A, ADRA1D | -97.6 |
| L-693403 | sigma receptor antagonist, sigma receptor ligand | PARP1 | -97.57 |
| warfarin | cytochrome P450 inhibitor, vitamin inhibitor, vitamin K epoxide reductase inhibitor | DRD2, SIGMAR1 | -97.43 |
| olanzapine | dopamine receptor antagonist, serotonin receptor antagonist | VKORC1, CYP2C19, CYP2C8, CYP4F2 | -97.39 |
| immethridine | histamine receptor agonist | DRD2, HTR2A, HTR2C, DRD1, DRD3, DRD4, HRH1, HTR1A, HTR1B, HTR1D, HTR1E, HTR6, HTR7, ADRA1A, ADRA1B, ADRA2A, ADRA2B, ADRA2C, ADRB1, ADRB2, ADRB3, CHRM1, CHRM2, CHRM3, CHRM4, CHRM5, CYP2C8, DRD5, GABRA1, GABRA2, GABRA3, GABRA4, GABRA5, GABRA6, GABRB1, GABRB2, GABRB3, GABRD, GABRE, GABRG1, GABRG2, GABRG3, GABRP, GABRQ, HRH2, HRH4, HTR1F, HTR2B, HTR3A, HTR5A | -97.22 |
| ST-91 | adrenergic receptor agonist | HRH3 | -97.03 |
| retinol | RAR receptor binder |  | -97.01 |
| eugenitol | androgen receptor (AR) inhibitor, free radical scavenger, monoamine oxidase inhibitor, quorum sensing signaling modulator | ALDH1A1, ALDH1A2, ALDH1A3, DHRS3, DHRS4, LRAT, NR2C2, RBP1, RBP3, RDH11, RDH12, RDH13, RDH14, RDH5, RDH8, RETSAT, RHO, RLBP1, RXRA, RXRB, RXRG | -97 |
| bosentan | endothelin receptor antagonist | AR, MAOA | -96.85 |
| ARC-239 | adrenergic receptor antagonist | EDNRB, EDNRA, ABCB1, CYP2C19, CYP3A4 | -96.82 |
| NECA | adenosine receptor agonist | ADRA2B, ADRA2A, ADRA2C | -96.68 |
| sirolimus | mTOR inhibitor, CCR expression inhibitor, cell cycle inhibitor, proteasome inhibitor, protein kinase inhibitor, T cell inhibitor | ADORA1, ADORA2A, ADORA2B, ADORA3, HSP90B1 | -96.66 |
| doxylamine | histamine receptor antagonist | MTOR, FKBP1A, CCR5, FGF2 | -96.51 |
| SB-525334 | TGF beta receptor inhibitor | HRH1, CHRM1 | -96.47 |
| lansoprazole | ATPase inhibitor | TGFBR1 | -96.44 |
| BRD-K41143549 | glutamate receptor antagonist | ATP4A, CYP2C19, CYP3A4, CYP3A5 | -96.41 |
| naltrexone | opioid receptor antagonist, opioid receptor ligand |  | -96.31 |
| tyrphostin-AG-1295 | FLT3 inhibitor, PDGFR tyrosine kinase receptor inhibitor | OPRK1, OPRM1, OPRD1, SIGMAR1 | -96.16 |
| GANT-58 | GLI antagonist | FLT3, KDR, PDGFRA, PDGFRB | -96.14 |
| deoxycholic-acid | biliverdin reductase A activator, G protein coupled receptor agonist, unidentified pharmacological activity | DHH, GLI1, IHH | -96.09 |
| PHTPP | estrogen receptor antagonist | GPBAR1, EFTUD1, FPR1 | -95.92 |
| VX-222 | HCV inhibitor, RNA-directed RNA polymerase inhibitor | ESR2 | -95.85 |
| geranylgeraniol | farnesyltransferase inhibitor |  | -95.72 |
| etomoxir | carnitine palmitoyltransferase inhibitor, carnitine O-palmitoyltransferase inhibitor, fatty acid oxidation inhibitor |  | -95.66 |
| nicergoline | adrenergic receptor antagonist | CPT1A, CPT1B | -95.44 |
| TTNPB | RAR agonist, retinoid receptor agonist | ADRA1A | -95.43 |
| propantheline | acetylcholine receptor antagonist | RARB, RARA, RARG | -95.4 |
| PRL-3-inhibitor-I | PRL phosphatase inhibitor, tyrosine phosphatase inhibitor | CHRM1, CHRM2, CHRM3, CHRM4 | -95.28 |
| enalaprilat | angiotensin converting enzyme inhibitor | PTP4A3 | -95.28 |
| BRD-K87426499 | caspase inhibitor | ACE | -95.22 |
| etodolac | cyclooxygenase inhibitor, TRPV agonist | CASP3 | -95.21 |
| QX-314 | sodium channel blocker | PTGS2, PTGS1, RXRA, TRPV1 | -95.16 |
| TCPOBOP | constitutive androstane receptor (CAR) agonist | MAPK14, TGFBR1 | -95.13 |

## 1.4 Table S4. The raw values of in vivo assays.

Weight of the tumor (21days, in grams, g)

| \| CTRL \| 0.5 \| 0.501 \| 0.56 \| 0.44 \| 0.51 \| \| --- \| --- \| --- \| --- \| --- \| --- \| \| shCHI3L2 \| 0.1 \| 0.14 \| 0.14 \| 0.11 \| 0.36 \| \| shFSTL3 \| 0.14 \| 0.11 \| 0.11 \| 0.12 \| 0.17 \| \| shRPA3 \| 0.11 \| 0.11 \| 0.11 \| 0.12 \| 0.18 \| \| shRRM2 \| 0.11 \| 0.11 \| 0.11 \| 0.17 \| 0.11 \| \| shYTHDF2 \| 0.14 \| 0.11 \| 0.12 \| 0.13 \| 0.13 \| |
| --- | --- | --- | --- | --- | --- | --- | --- | --- | --- | --- | --- | --- | --- | --- | --- | --- | --- | --- | --- | --- | --- | --- | --- | --- | --- | --- | --- | --- | --- | --- | --- | --- | --- | --- | --- | --- |
